# Structure-based design of glycoprotein subunit vaccines for mumps

**DOI:** 10.1073/pnas.2404053121

**Published:** 2024-11-11

**Authors:** Rebecca J. Loomis, Yen-Ting Lai, Sun B. Sowers, Brian Fisher, Alexandrine Derrien-Colemyn, David R. Ambrozak, Yaroslav Tsybovsky, Stephen N. Crooke, Donald R. Latner, Wing-Pui Kong, Tracy J. Ruckwardt, Stanley A. Plotkin, Peter D. Kwong, John R. Mascola, Barney S. Graham, Carole J. Hickman, Guillaume B. E. Stewart-Jones

**Affiliations:** ^a^Vaccine Research Center, NIH, Bethesda, MD 20892; ^b^Division of Viral Diseases, National Center for Immunization and Respiratory Diseases, Centers for Disease Control and Prevention, Atlanta, GA 30333; ^c^Electron Microscopy Laboratory, Cancer Research Technology Program, Frederick National Laboratory for Cancer Research sponsored by the National Cancer Institute, Frederick, MD 21701; ^d^Department of Pediatrics, University of Pennsylvania, Doylestown, PA 18902

**Keywords:** mumps, subunit vaccine, structure-based design, paramyxovirus, glycoprotein

## Abstract

Mumps virus (MuV) is a highly contagious paramyxovirus that is endemic in most regions of the world and continues to cause outbreaks even in highly immunized populations. Outbreaks of mumps in countries with high measles, mumps, and rubella vaccination coverage have been attributed to waning immunity and antigenic differences between the Jeryl Lynn vaccine strain (genotype A) and circulating wild-type viruses. To obtain a subunit vaccine, we used structure-based design to engineer the mumps fusion (F) glycoprotein stabilized in its prefusion conformation (Pre-F) as well as a chimeric immunogen comprising Pre-F linked to mumps hemagglutinin neuraminidase (HN); in mice, both Pre-F antigen and the chimeric antigen elicited potent cross-reactive plaque reducing neutralizing titers to genotypes A, G, and H mumps. A crystal structure of mumps Pre-F at 2.16 Å resolution validated the stabilization strategy, while a post-fusion form of F was engineered as a comparator. Monoclonal antibodies to mumps Pre-F and HN were isolated from immunized mice; 7 of 14 Pre-F-specific antibodies and 9 of 15 HN-specific antibodies were capable of neutralizing genotype G MuV with a range of potencies. Additionally, 7 of 14 Pre-F-specific antibodies neutralized genotype A mumps. Structural and binding analyses of Pre-F-specific antibodies revealed binding to four discrete neutralizing antigenic sites and binding analyses of HN-specific antibodies revealed binding to five discrete neutralizing antigenic sites. Overall, the PreF and the chimeric Pre-F/HN immunogens are promising candidates to boost MMR-elicited immunity to mumps or as a next-generation vaccine.

## Significance

Mumps is typically a childhood illness that can have serious medical consequences, including parotitis, orchitis, meningitis, encephalitis, and deafness. Since 2006, there has been a resurgence of cases of mumps disease in the United States of America among highly vaccinated populations, with >18,000 affected individuals. Contributing factors may include waning immunity and antigenic differences between vaccine and circulating wild-type strains that may limit antibody effectiveness. Structural engineering of F and HN glycoproteins has led to the identification of prefusion-stabilized F and chimeric immunogens that elicit high-titer neutralizing antibodies to diverse mumps viruses in mice compared to titers routinely observed following MMR vaccination in humans. These Pre-F and Pre-F/HN chimeric glycoproteins are promising primary series or booster vaccine candidates for mumps.

Before the introduction of mumps-containing vaccines in the late 1960s, mumps caused widespread morbidity characterized by fever, parotitis and less commonly, orchitis, meningitis, encephalitis, and deafness (1–3). The live-attenuated vaccine for mumps and subsequent combination measles, mumps, and rubella vaccine (MMR) dramatically reduced the incidence of mumps throughout the world (4, 5). Two doses of MMR vaccine are approximately 88% effective for the prevention of mumps disease. However, since 2006, there has been a resurgence in the number of cases of mumps disease in the world among highly vaccinated populations, with >18,000 affected individuals in the United States of America (6–10). The reasons for this are not fully understood, but contributing factors may include waning immunity (11, 12), variability in antibody responses (13), and antigenic differences between the vaccine and circulating wild-type strains (14). The predominant mumps genotype associated with outbreaks in the United States and Europe in recent years has been genotype G (15, 16). Mumps outbreak studies have shown that a threshold titer of 1:8 against Iowa-G/USA-06 was able to distinguish cases from noncases (17) and a level of neutralizing antibody of 1:16 for genotype G virus correlated with protection from clinical mumps disease during a 2017 mumps outbreak among military service members (18). While neutralizing antibody is considered a biomarker of immunity to mumps (19), to date, an immunological correlate of protection has not been formally defined.

The mumps live-attenuated viral vaccine was developed over 55 y ago by attenuation through repeated passage in heterologous cells and tissues (20). However, recent advances in antigen characterization and engineering allow for more precise approaches to vaccine design, particularly for class I fusion glycoproteins of pneumoviruses and paramyxoviruses as demonstrated by the recent launch of recombinant protein prefusion-stabilized F vaccines for respiratory syncytial virus (RSV) (21–23). Here, we report the design, crystal structure to 2.16 Å resolution of a mumps genotype G Pre-F glycoprotein trimer, and the engineering of a chimeric mumps Pre-F trimer fused with mumps genotype G HN head domain (Pre-F/HN). We evaluated these designs as immunogens in mice by measuring the neutralizing activity of sera and cloned murine monoclonal antibodies. We structurally mapped vaccine-elicited antibodies bound to the prefusion mumps F glycoprotein trimer using negative-stain electron microscopy (NS-EM) and competition binding studies and identified four distinct neutralizing sites that differentially neutralize mumps genotypes G and A. By coupling plaque reduction neutralization test (PRNT) results with competition binding studies, we mapped vaccine-elicited HN-specific antibodies to five distinct neutralizing sites, active to both mumps genotype G and A. These immunogens developed through structure-based design have the potential to become clinical candidates to prevent complications of mumps infection.

## Results

### Design of a Disulfide Bond and Membrane-Proximal Coiled Coil Stabilized Glycoprotein with Enhanced Yield of the Soluble Prefusion Mumps F Trimer.

Neutralization titers elicited by paramyxovirus Pre-F glycoproteins are more potent than those elicited by their Post-F counterparts (22–24); therefore, we aimed to develop a prefusion-stabilized mumps F glycoprotein trimer. We observed from NS-EM that purified native mumps F glycoprotein with only the GCN4 trimerization motif on the C-terminal coiled coil was primarily in the Post-F conformation. We used the crystal structures of the prefusion parainfluenza virus 5 (PIV5) F glycoprotein (PDB IDs 4GIP, 4WSG) (25) to construct a homology model for the prefusion mumps F protein, consisting of three intertwined monomers forming a quaternary assembly of DI, DII, DIII, and HRB domains. By creating a matrix of disulfide substitutions and C-terminal coiled-coil-GCN4 attachment positions while mutating cleavage site residues 101–103 to three glycine residues (Gly3), we evaluated the level of protein expression and the proportion of protein adopting the prefusion or postfusion conformation using NS-EM (*SI Appendix*, Fig. S1 *A* and *B*). While five combinations of disulfide bonds, cleavage site mutations, and GCN4 attachment positions gave rise to 100% prefusion trimers, only one such combination resulted in high-yield protein expression (V206C-A223C with 476-GCN4 and 101KRF-101GGG) at about 4.8 mg/L from Expi293 cells (*SI Appendix*, Fig. S1*A*). The NS-EM 2D average of this design (*SI Appendix*, Fig. S1 *B*, *Top Inset*) contrasts with the unstabilized 476-GCN4 mumps Post-F glycoprotein trimer (*SI Appendix*, Fig. S1 *B*, *Lower Inset*) consistent with prior observations of paramyxovirus F protein conformations (22–28). We focused on the highest-expressing Pre-F design and carried out further characterization and immunization studies to assess it as a vaccine candidate.

### Crystal Structure of Mumps Pre-F Glycoprotein Trimer at 2.16 Å Resolution Reveals a Stabilizing Disulfide Bonded Design and the Location of Polymorphic Residues.

**Fig. 1.**
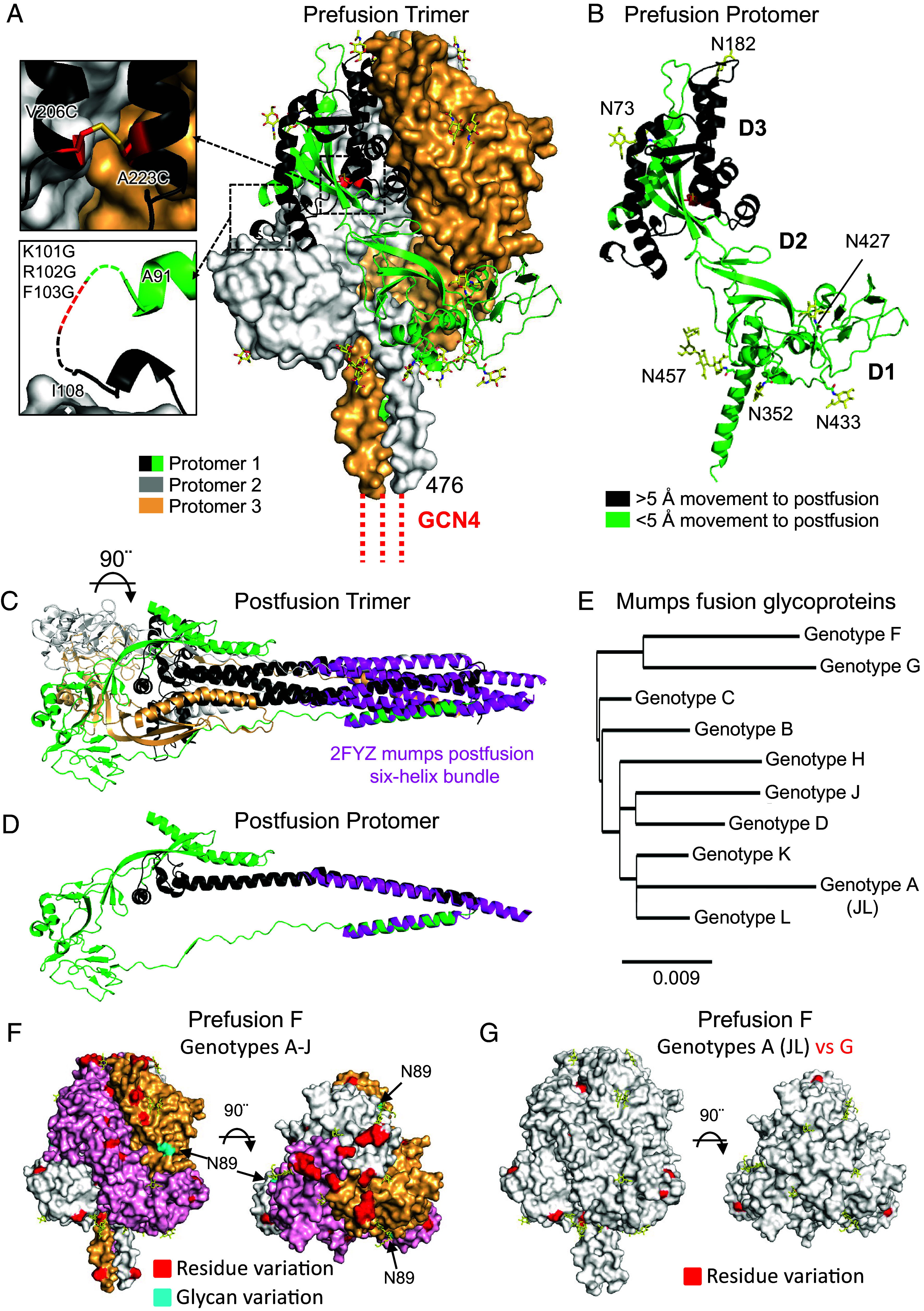
Structure-based design of a disulfide stabilized prefusion mumps F trimer. (*A*) Crystal structure to 2.16 Å resolution of prefusion mumps F trimer with protomers shown in white, light orange, green, and black where residues undergo >5 Å conformational change to transition to the postfusion conformation. GCN4 trimerization motif is shown in red dash, connected to F residue 476. Zoom insets highlight mutated residues (red) to stabilize the trimeric prefusion F structure. (*B*) A single protomer of the prefusion mumps trimer showing the D1–D3 subregions and the locations of the six *N*-linked glycans (yellow) per protomer. (*C*) Corresponding postfusion F mumps trimer model based on postfusion PIV3 F, colored according to (*A*), orientated 90° clockwise to the prefusion trimer in (*A*), also aligned to the crystal structure of mumps postfusion F 6-helix bundle 2FYZ (magenta). (*D*) A single protomer of the postfusion mumps trimer aligned to a single protomer of the mumps postfusion 6-helix bundle (magenta). (*E*) Phylogenetic analysis of mumps genotypes A-L fusion glycoprotein sequences. (*F*) Structural mapping of mumps genotype variations on prefusion F showing A-J genotype variation and (*G*) genotype G versus A [Jeryl Lynn (JL)], showing residue differences (red) and glycan differences (cyan).

Size-exclusion chromatography of mumps Pre-F revealed a single peak (*SI Appendix*, Fig. S1*C*) which, when expressed in the presence of kifunensine and the 6 potential N-linked glycosylation sites (PNGS) per protomer enzymatically deglycosylated (Pre-F-Δglycan, *SI Appendix*, Fig. S1*C*), could be crystallized. The x-ray structure to 2.16 Å resolution (Fig. 1 *A* and *B* and *SI Appendix*, Table S1 and Fig. S1 *D–F*) revealed a similar overall architecture to PIV5 and other paramyxovirus Pre-F trimers (22, 24–28). However, the apex of the mumps prefusion F trimer adopts a “closed cap” structure comprising loops N177-S184 formed by interactions between side chains T178, Q179, and N181, which has not been observed in other paramyxovirus Pre-F structures (*SI Appendix*, Fig. S1*E*) (22, 24–28). The remaining *N*-acetyl glucosamine moieties from the six glycans at positions N73, N182, N352, N427, N433, and N457 were visible in the electron density maps and a Man5 glycosylated model built (*SI Appendix*, Fig. S1*F*). The V206C-A223C disulfide was clearly defined by the electron density with a Cα–Cα atomic distance of 4.8 Å, whereas in the homologous postfusion PIV3 F trimer structure [PDB ID 1ZTM], Cα–Cα atoms are positioned 5.8 Å apart (29). The DI–DIII domains enclosed a large aqueous cavity measuring ~17,000 Å^3^ in volume and protomers bury a 6,500 Å^2^ interface, similar to that observed in PIV5, PIV3, Nipah, measles, and Langya Pre-F structures (22, 24–28).

While the sequence identities of mumps F and HN are relatively high between genotypes (Fig. 1*E* and *SI Appendix*, Fig. S1 *G* and *H*), we mapped the polymorphic variation to the structures of mumps Pre-F and HN (Fig. 1 *F* and *G* and *SI Appendix*, Fig. S1 *H* and *I*). Mapping the variation to Pre-F for all genotypes and specifically between genotype G and Jeryl Lynn (genotype A), much of the protein surface exposed for antibody recognition was conserved (Fig. 1 *F* and *G*), while numerous variable amino acids were located within the aqueous cavity in the core of the prefusion trimer. In contrast, the mumps HN dimer structures (30) revealed that most polymorphic amino acids are exposed to the solvent, including a glycan variation at position N464 between Jeryl Lynn and genotype G HNs (*SI Appendix*, Fig. S1 *H* and *I*). The predominance of solvent-exposed polymorphic residues on HN versus Pre-F may suggest resistance to genotype G mumps infection in those vaccinated with Jeryl Lynn may be more heavily influenced by variability in HN.

### Mumps Pre-F and Pre-F/HN Chimeric Antigens Elicit High-Titer Neutralizing Antibodies in Mice.

**Fig. 2.**
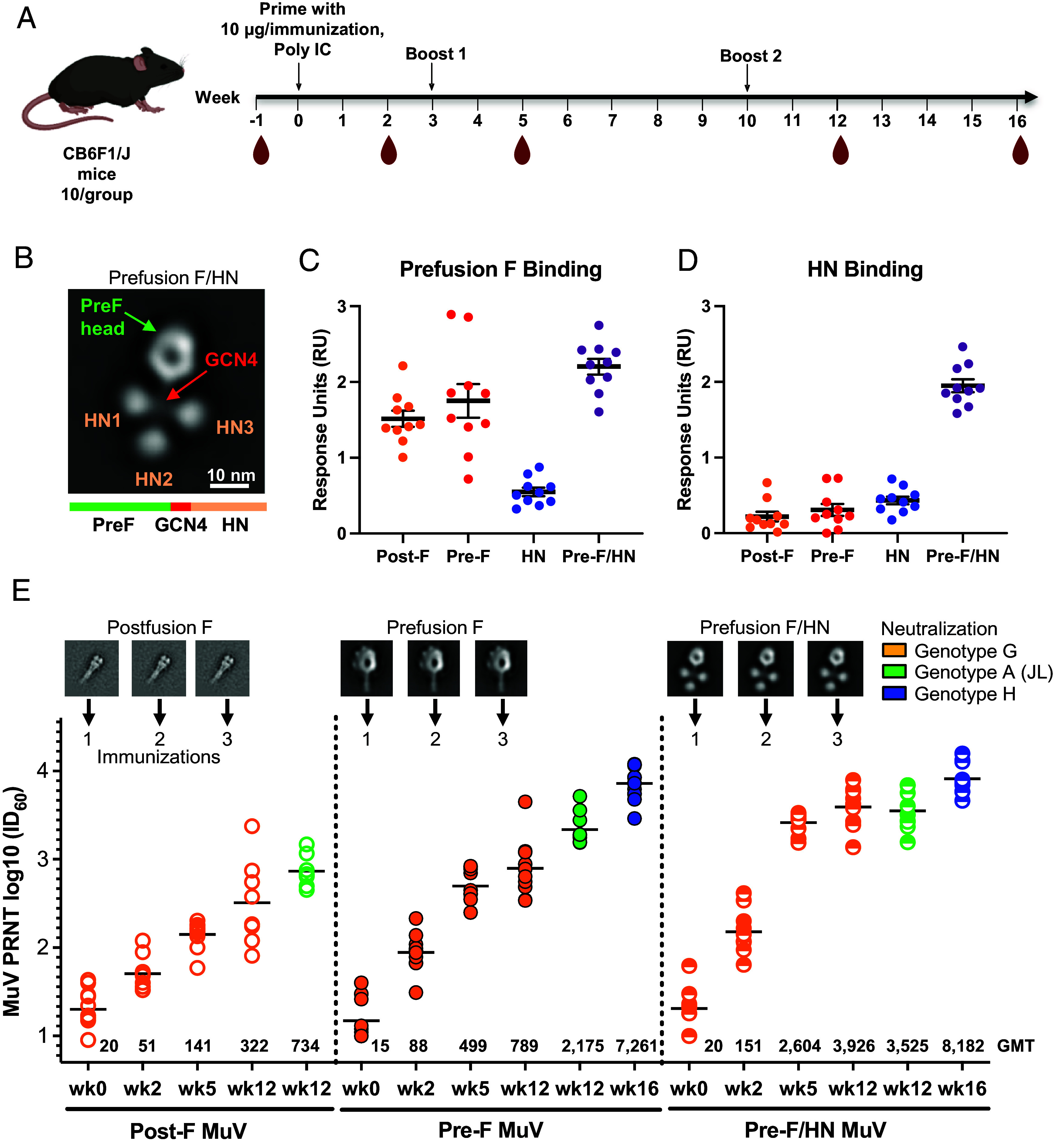
Immunogenicity of postfusion F, prefusion F and Pre-F/HN mumps vaccine designs. (*A*) Immunization in CB6F1/J mice was performed three times at 10 µg/dose with Poly I:C adjuvant at times 0, 3, and 10 wk with blood sampling at 2, 5, 12, and 16 wk for evaluation with BLI and plaque reduction neutralization (PRNT) assays. (*B*) Negative-stain characterization of mumps Pre-F/HN design, showing structures for the Pre-F trimer, GCN4 trimerization domain and three HN globular head domains. Octet biointerferometry binding titers for immunized mice using (*C*) the mumps prefusion F probe or (*D*) the HN globular head domain probe. (*E*) Mumps PRNT titers for postfusion F immunogen (*Left*, open circles), prefusion F immunogen (*Center*, solid circles), and prefusion F/HN immunogen (*Right*, top-half filled circles), after three immunizations at weeks 0, 3, and 10 in mice (shown with arrows), and PRN titers for genotype G virus (orange), Jeryl Lynn genotype A virus (green), and genotype H virus (blue).

We evaluated the ability of mumps Pre-F to elicit neutralizing antibodies compared to other mumps immunogens. CB6F1 mice (10 per group) were immunized with polyI:C-adjuvanted mumps glycoproteins at weeks 0, 3, and 10 (Fig. 2*A*). In addition to mumps Pre-F and Post-F, we tested monomeric HN and the chimeric immunogen comprising mumps Pre-F C-terminally linked with genotype G HN soluble headgroup (Pre-F/HN) (*SI Appendix*, Table S2), a design that we showed in prior work with Nipah vaccine candidates elicited antibodies to both surface glycoproteins (24). This Pre-F/HN design yielded approximately 0.3 mg/L from Expi293 cells, was monodispersed on size exclusion chromatography, and displayed expected assembly from NS-EM (Fig. 2*B* and *SI Appendix*, Fig. S1*C*). While Fig. 2*B* shows a representative two-dimensional class average of the Pre-F/HN design, multiple different arrangements of the HN headgroup relative to the PreF moiety were observed under EM, owing to the flexible linker utilized between the GCN4 motif and the HN headgroup.

Using bio-layer interferometry (BLI), antibody responses to Pre-F were detected in sera of mice immunized with mumps Pre-F-containing immunogens (Pre-F or Pre-F/HN), with slightly lower levels of binding to sera from Post-F immunized mice, although not statistically significant compared to sera from Pre-F immunized mice (Fig. 2*C*). Recombinant HN monomer bound only HN-immunized mouse sera, however, monomeric HN-immunized mice showed little binding (also with a low genotype G geometric mean PRNT ID_60_ value of 1:119 after three doses), whereas sera from Pre-F/HN-immunized mice showed substantial levels of HN binding suggesting multivalency of HN drives a robust humoral responses (Fig. 2*D*).

To evaluate the elicitation of neutralizing antibody, we measured the genotype G PRNT in mice immunized with mumps Post-F, Pre-F, and Pre-F/HN (Fig. 2*E*). Increases in neutralizing activity were observed after each immunization, however, the third immunization marginally increased neutralization titers compared to the second (Fig. 2*E*). Neutralizing antibodies were observed following Post-F immunization, however, the Pre-F elicited 3.5- and 2.5-fold higher neutralization than Post-F after the second [geometric mean inhibitory dose (ID_60_ value) of 141 and 499, respectively] and third immunization (geometric mean ID_60_ values) of 322 and 789, respectively, for genotype G mumps virus (MuV), respectively. After the third immunization, the Pre-F/HN chimeric protein elicited neutralizing titers 12-fold higher than Post-F and 5-fold higher than Pre-F, with an geometric mean ID_60_ value of 3,926 to genotype G virus (Fig. 2*E*). The Pre-F/HN chimera elicited higher neutralizing activity than Pre-F alone (comparing Pre-F and Pre-F/HN post two doses, in an unpaired *t* test, *P*-value < 0.0001; Fig. 2*E*).

We further evaluated the PRNT to genotype A (Jeryl Lynn) and genotype H viruses to characterize cross-neutralizing antibodies elicited by the engineered immunogens. For Post-F and Pre-F, we observed a greater level of neutralization elicited to Jeryl Lynn virus than to genotype G virus, whereas for the Pre-F/HN chimera, equivalent PRNTs were observed. At week 16, sera from both Pre-F and Pre-F/HN groups showed robust PRNT to genotype H virus, confirming that these recombinant immunogens can elicit antibodies that can cross-neutralize three divergent genotypes of mumps (Fig. 2*E*). We assessed the durability of neutralizing activity to genotypes A, G, and H MuVs for 6 mo after the third dose and found that while titers naturally wane, ID_60_ stabilize after three months. Pre-F-immunized mice had geometric mean PRNT for genotype G, Jeryl Lynn, and genotype H viruses at approximately 100, 800, and 3,200, respectively, after 6 mo (*SI Appendix*, Fig. S2*A*). In comparison, ID_60_ PRNT of sera from mice immunized with the Pre-F/HN remains stable at approximately 750, 900, and 4,300 for genotypes G, Jeryl Lynn, and genotype H viruses, respectively, 6 mo after the third dose (*SI Appendix*, Fig. S2*B*). Overall, mumps Pre-F/HN yielded neutralization titers higher than F alone, demonstrating that the chimeric design strategy enables the elicitation of responses to both mumps surface antigens and elicits potent neutralizing activity from a single polypeptide immunogen.

### Analysis of Vaccine-Elicited Monoclonal Antibodies Reveals Diverse Pre-F and HN Responses.

**Fig. 3.**
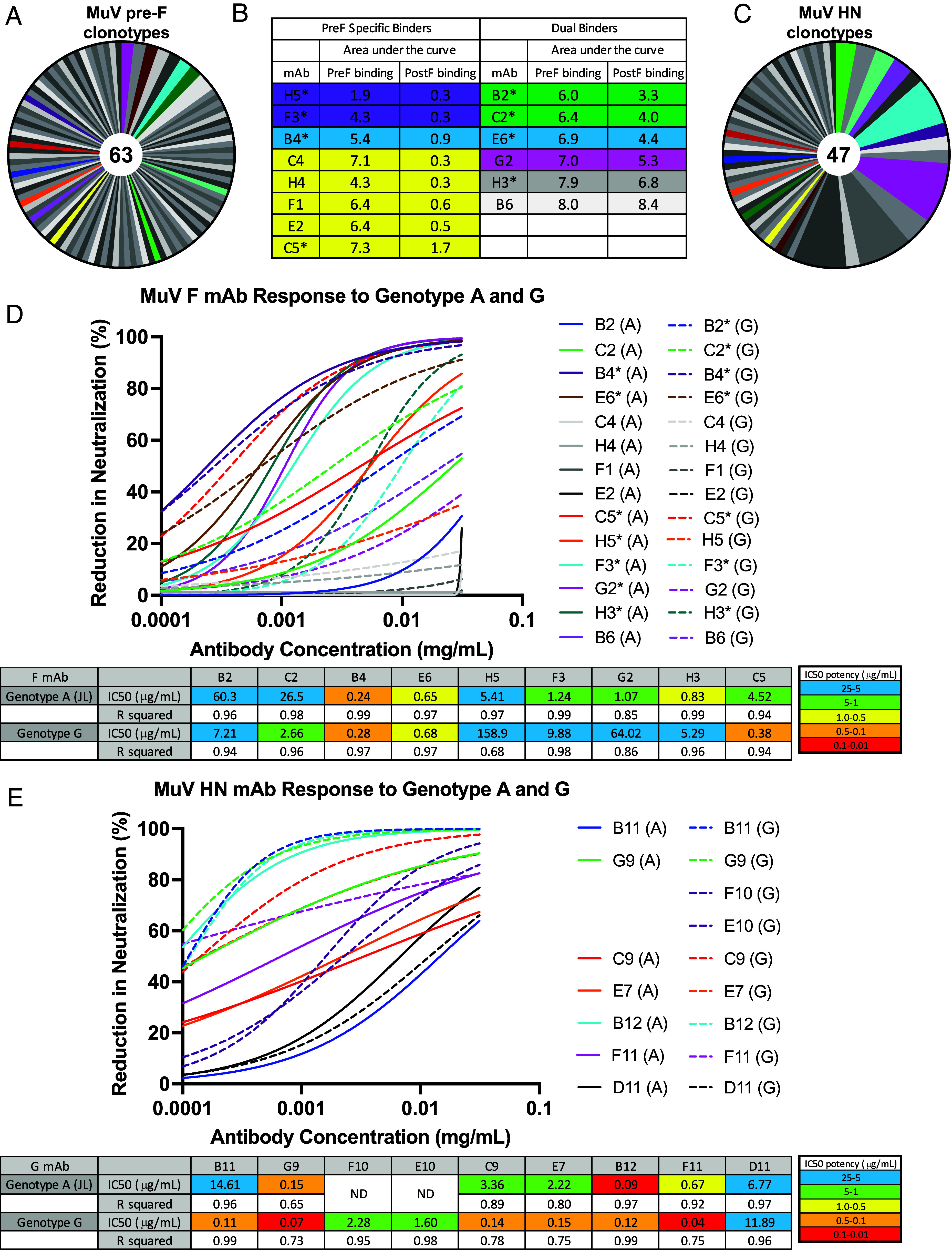
Characterization of mumps prefusion F specific monoclonal antibodies from mice and antibody epitope cross-competition binding with negative-stain EM structures of mAb-preF complexes. (*A*) 63 unique murine clonotypes were identified from paired antibody heavy and light chain sequenicing using a prefusion F probe to sort B cells from prefusion F immunized mice. The frequency of these clonotypes is shown in the pie charts. 32 F specific murine MuV mAb clonotypes were synthesized, expressed, and evaluated for binding to Pre-F protein. (*B*) ELISA binding to prefusion and postfusion F protein for expressed monoclonal antibodies. Asterisks indicate antibodies that were shown to be neutralizing via PRNT. (*C*) 47 unique murine clonotypes were identified with a HN probe to sort B cells from prefusion F/HN immunized mice. The frequency of these clonotypes is shown in the pie charts. 21 HN specific murine MuV mAb clonotypes were synthesized, expressed, and evaluated for binding to pre-F or HN protein. (*D*) Characterization of mumps prefusion F specific monoclonal antibodies from mice. (*Top* panel) The highest binding 14 mAbs to pre-F were evaluated in PRNT with genotype G and genotype A (JL) virus and IC_50_ neutralization potencies determined. (*Bottom* panel) 9/14 preF specific antibodies were capable of neutralizing genotype G virus or genotype A virus (Jeryl Lynn) with a range of potencies from 0.24 μg/mL to 160 μg/mL. (*E*) Characterization of mumps HN specific monoclonal antibodies from mice. (*Top* panel) The highest binding mAbs to HN were evaluated in PRNT with genotype G and genotype A (Jeryl Lynn) virus and IC_50_ neutralization potencies determined. (*Bottom* panel) 9/15 HN specific antibodies were capable of neutralizing genotype G virus with a range of potencies and 7/15 HN specific antibodies were capable of neutralizing genotype A virus with a range of potencies.

Following an initial immunization series (weeks 0, 3, and 10) as described in Fig. 2*A*, CB6F1/J mice were boosted at week 18 with either mumps Pre-F or mumps Pre-F/HN. The time between the initial immunization series and the boosts used for B cell sorting allowed for maturation and increased affinity of the antibody response. We generated biotinylated Pre-F and monomeric HN probes for single B cell sorting and sequencing. Isolated splenocytes were screened using an established antibody panel to identify B cells specific to the included mumps prefusion F-conjugated probe or mumps HN-conjugated probe, which were single-cell index-sorted by fluorescence-activated cell sorting (FACS) into 96-well plates (*SI Appendix*, Fig. S3 *A*–*F*). The B cell receptor of sorted B cells were sequenced to obtain paired heavy and light chain sequences (*SI Appendix*, Table S3). Sixty-three Pre-F-specific and 47 HN-specific paired heavy- and light-chain sequences were obtained from Pre-F and Pre-F/HN immunized mice, respectively (Fig. 3 *A* and *C*). Thirty-two unique Pre-F-specific clonotypes and 21 unique HN-specific clonotypes were identified, expressed, and tested for antigen binding. Fourteen genetically diverse F and fifteen HN monoclonal antibodies (*SI Appendix*, Tables S4 and S5) were selected for production and tested for neutralizing activity to genotype G mumps (Fig. 3 *D* and *E*). Of the 29 mAbs tested for potency, 9/14 F and 9/15 HN-directed mAbs had IC_50_ values ranging from 0.05 µg/mL to 160 µg/mL to genotype G or genotype A with two HN-specific antibodies showing the highest potency around 0.05 μg/mL (Fig. 3 *D* and *E*).

**Fig. 4.**
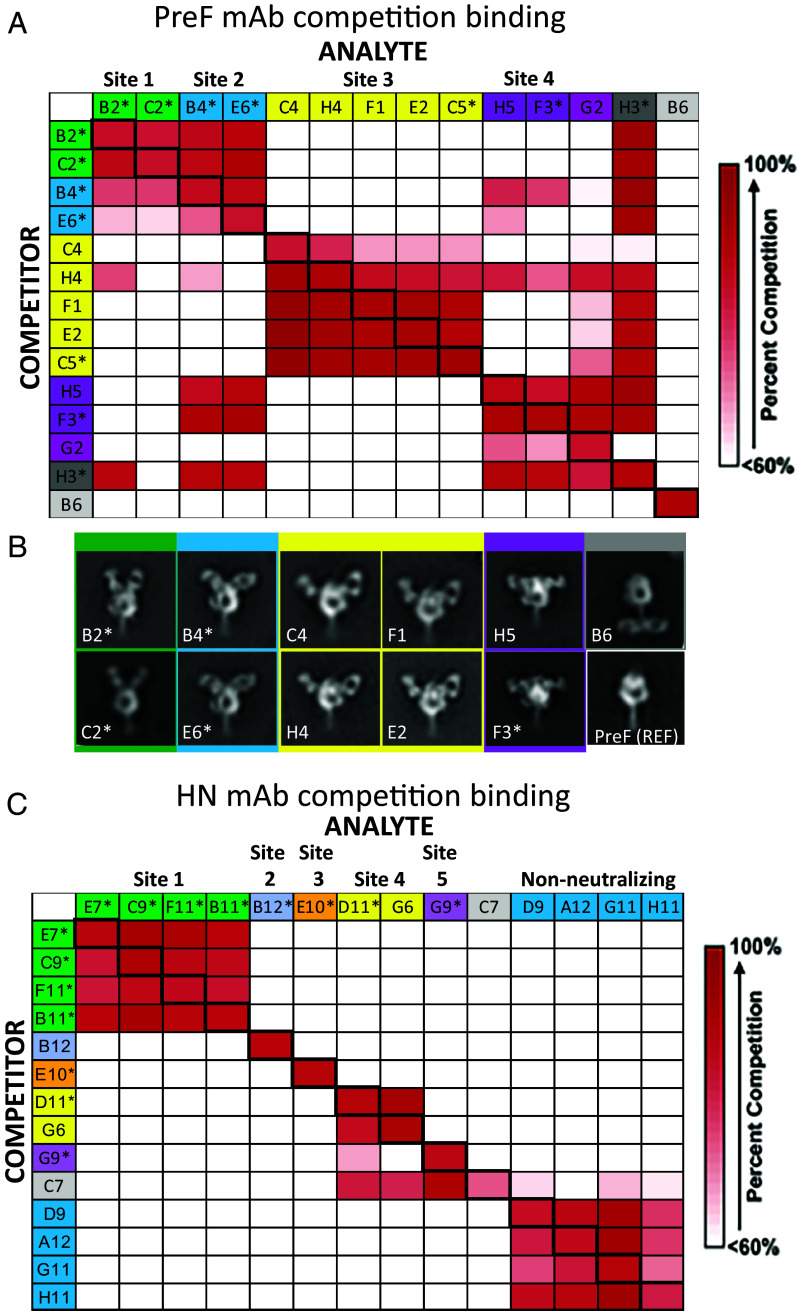
(*A*) Antibody competition table showing percentage inhibition of binding mAbs (column) to immobilized preF saturated with competing antibody (row) using Octet interferometry. Antibodies are arranged in competition groups and asterisks indicate which antibodies are neutralizing. (*B*) Negative-stain EM of prefusion mumps F bound to mAbs arranged into competition groups with neutralizing antibodies shown with an asterisk. Four distinct neutralizing epitopes (antigenic sites 1 to 4) were identified on the membrane-distal region of the prefusion mumps F glycoprotein trimer. Nonneutralizing antibodies (with the exception of B6, which binds the C-terminal coiled coil of the molecule) appear to bind prefusion mumps F in similar modes as neutralizing antibodies. The unbound prefusion F trimer is shown in the *Lower Right* panel for reference. Antigenic sites 1 to 4 are ordered based on the mAb binding angle relative to the F trimer axis. (*C*) Antibody competition table showing percentage inhibition of binding mAbs (column) to immobilized HN saturated with competing antibody (row) using Octet interferometry. Antibodies are arranged in competition groups and asterisks indicate which antibodies are neutralizing.

Competition BLI was used to cluster Pre-F-specific antibodies into groups targeting distinct overlapping or nonoverlapping epitopes on mumps Pre-F (Fig. 4*A*). Based on the competition patterns, four groups of Pre-F antibodies were found to compete with each other while one antibody did not exhibit a clear competition pattern. NS-EM analysis of Fab-Pre-F complexes revealed a diverse set of antibody-antigen binding modalities (Fig. 4*B*), ranging from apical binders (B2 and C2) to more angular binders (B4, E6, C4, H4, F1, E2) and two lateral binders (H5 and F3). The NS-EM results corroborated the competition binding data, for example, antibodies B2 and C2 bound similarly at the apex of the Pre-F structure and they competed; while antibodies F3 and H5 exhibited similar binding modes and had comparable competition profiles (Fig. 4 *A* and *B*). This correspondence between EM analysis and competition data applied to all identified antibodies including B6, which bound at the lower portion of the stem and did not compete with any other antibody. It is notable that all 14 Pre-F antibodies selected for detailed characterization, with the exception of the stem binding antibody B6, bind membrane-distal epitopes of the Pre-F trimer, which is predicted to undergo conformational changes to transition to Post-F (Fig. 1 *A*–*D*). Potently neutralizing antibodies targeting the membrane-distal epitopes have been similarly observed for other viral fusion glycoproteins, such as RSV F (31) and PIV3 F (22, 23). The antibody competition and structural and neutralization analyses allowed the definition of four distinct mumps neutralizing F-specific epitopes (mumps F antigenic sites 1 to 4) on the membrane-distal region of the mumps Pre-F (Fig. 4 *A* and *B*).

Five distinct HN-specific neutralizing antibody sites were identified based on competition binding patterns (Fig. 4*C*). Unlike Pre-F specific neutralizing antibodies, HN neutralizing antibodies had limited overlapping binding sites. Interestingly, four nonneutralizing antibodies (D9, A12, G11, H11) clustered and competed with each other, implying an immunodominant nonneutralizing epitope on mumps HN which can potentially be masked in future engineering efforts to focus immune responses to neutralizing epitopes.

**Fig. 5.**
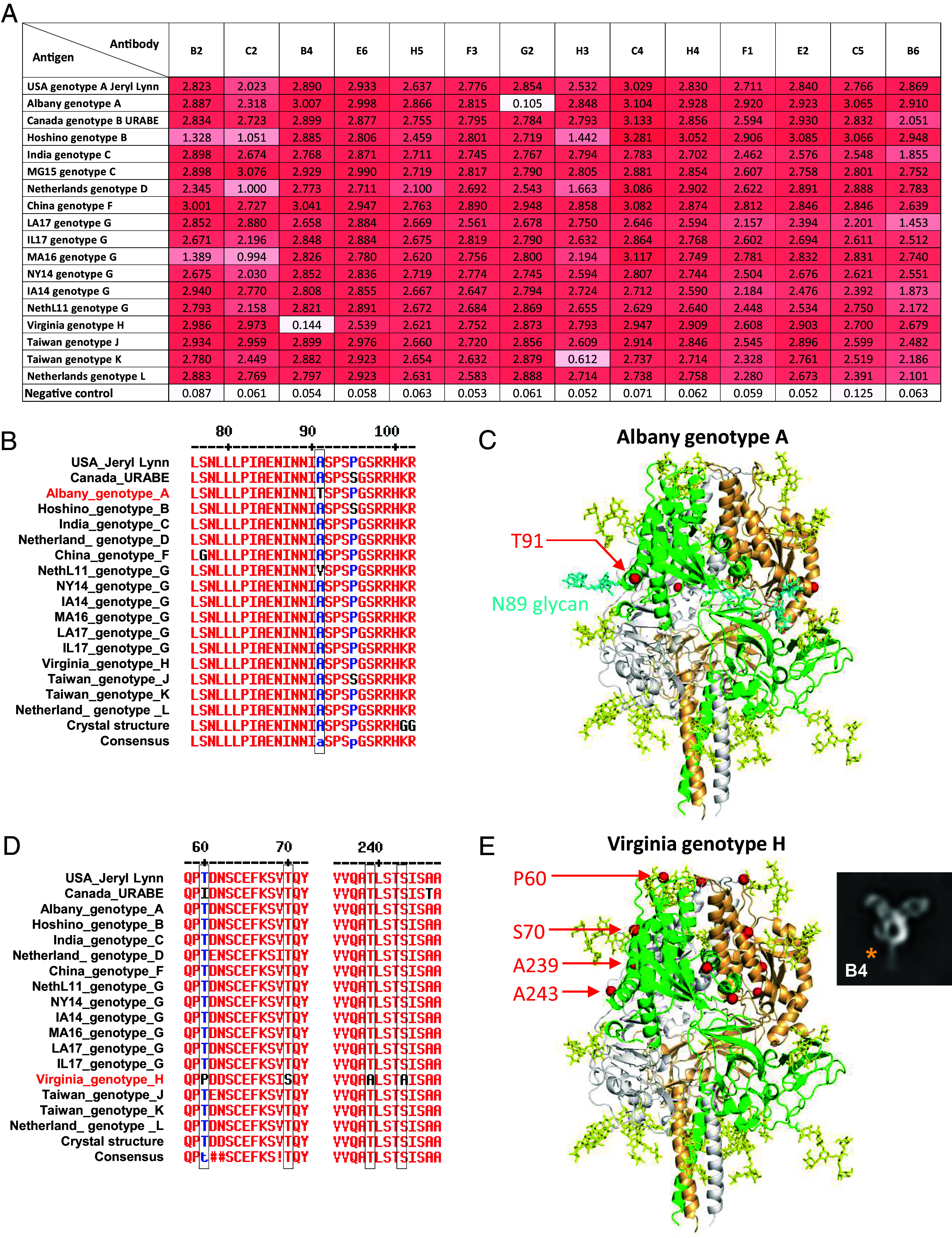
Cross-reactivity profiling reveals Pre-F specific murine mAbs are broadly cross-specific to diverse genotype F Pre-F protein trimers. (*A*) BLI binding showing broad cross-recognition of the vaccine-elicited mAbs to a diverse set of prefusion-stabilized F antigens from genotypes A–L. Occasionally, some strains are resistant to antibody binding, however the frequency is low. (*B*) Sequence alignment of F glycoproteins from diverse strains, highlighting a unique residue in the Albany genotype A and (*C*) structural mapping of the unique residue of Albany genotype A, revealing a glycan added to Pre-F which impacts antibody G2. (*D*) Sequence alignment of F glycoproteins from diverse strains, highlighting Virginia genotype H and (*E*) mapping unique residues of the Virginia genotype H strain onto the prefusion F trimer crystal structure, revealing that multiple residue differences are apparent in the region B4 antibody binds to the prefusion F site 2.

We evaluated the cross-reactive binding using BLI of the 14 Pre-F antibodies to 18 diverse genotypes of mumps Pre-F proteins, stabilized in their prefusion conformation using the V206C-A223C and 101KRF-101GGG mutations and addition of 476-GCN4 (Fig. 5*A*). We observed that 9 Pre-F-specific antibodies were cross-reactive to all the Pre-F genotypes tested (binding threshold of >1.45 to Pre-F). Some antibodies showed decreased or diminished binding to specific strains, for example, antibody G2 does not bind to the Albany genotype A strain F which could be mapped using modeling to a single residue change, T91 (alanine in other tested strains) and which introduces an *N*-linked glycosylation site at residue N89 (Fig. 5 *B* and *C*). This likely abrogated G2 binding, and given its unique antibody competition profile, the G2 epitope can be mapped to a region encompassing residues 89–91.

Antibody B4 displayed a loss of binding to the Virginia genotype H strain Pre-F, which has numerous unique amino acid changes compared to other tested strains (Fig. 5 *D* and *E*). Comparing the binding position of the B4 Fab relative to the Pre-F trimer in the EM analysis, the cluster of residues comprising S70, A239, and A243 are likely within the antibody binding footprint (Fig. 4*B*). However, antibodies E6 and H3, which compete with B4 to antigenic site 2, are unaffected by the variations in the Virginia genotype H strain Pre-F, suggesting that these antibodies bind with subtle differences to the site.

## Discussion

MMR vaccination in humans results in GMT IC_50_ PRNT around 220 for Jeryl Lynn and 35 for genotype G mumps (32). The cross-reactive high PRNT induced in mice by immunization with recombinant prefusion-stabilized mumps F glycoprotein immunogens indicates that Pre-F induces neutralizing titers more effectively than Post-F as has been previously demonstrated for RSV, Nipah, and PIV1-4 (21, 22, 24). We demonstrate the capacity of the Pre-F/HN chimera to elicit higher neutralizing activity than Pre-F alone which aligns with similar antigen designs for Nipah virus (24). While balanced Pre-F and HN-specific serum antibody titers are elicited from the Pre-F/HN antigen, a genetically diverse group of F and HN monoclonal antibodies are elicited, with Pre-F-specific antibodies engaging the glycoprotein trimer at a spectrum of different epitopes and angles of engagement. Competition mapping enabled the definition of four discrete neutralizing antigenic sites in the membrane-distal portion of the prefusion trimer and five discrete neutralizing antigenic sites in the HN protein. It is notable that while site 3 and site 4 neutralizing mAbs (C5 and F3 respectively) bind the membrane distal region of the Pre-F trimer, these are Pre-F specific while site 1 neutralizing mAbs (B2 and C2) bind the apex of Pre-F, yet are dual specific with Post-F. The apex of the mumps prefusion F trimer adopts a closed cap structure which is proximal to the apparent B2 and C2 mAb binding sites, therefore, the epitope for these mAbs may include or rely on the interprotomer interactions at the apex. Further work is needed to understand the relationship between antigenic site-specificity, structure, and the impact of glycosylation and genotypic variation on neutralization, similar to what was done for the Pre-F protein of RSV (33).

Mumps Post-F elicits relatively high neutralization titers, a phenomenon observed for some paramyxoviruses such as PIV-2 and PIV-4, but not for PIV-3, PIV-1, or Nipah Post-F (22, 24); it is therefore possible that neutralization-sensitive epitopes are preserved on both mumps Pre-F and Post-F. The NS-EM of the mumps prefusion-specific neutralizing monoclonal antibodies which bind to the membrane distal region of Pre-F, suggest that a substantial proportion of neutralization epitopes exist in domains that undergo conformational change in the fusion process of the mumps F glycoprotein.

Comparing responses in Pre-F immunized mice to those immunized with Pre-F/HN, the HN component elicits better neutralizing activity (Pre-F vs. Pre-F HN PRNT of 499 and 2,604, respectively, *P*-value < 0.0001 in an unpaired *t* test). Inclusion of Pre-F component with HN broadens the immune response and presents a greater diversity of antigenic sites. It is noteworthy that while most Pre-F antigenic sites have neutralizing and nonneutralizing directed mAbs, the HN antigenic site antibodies tend to be neutralizing or nonneutralizing depending on the antigenic site. Our findings are broadly consistent with previous work on Nipah virus, another paramyxovirus (24). The Nipah Pre-F/G chimera demonstrated superior elicitation of Nipah-specific Tfh and other effector T cells compared to G alone in both mRNA and protein platforms and to increase breadth and frequency of T cell responses (34). When coupled with the neutralizing antibody activity to both glycoproteins, the chimera had an advantage compared to each individual antigen. While we did not evaluate the antigen-specific T cell responses of our MuV designs, we cannot exclude a possible role for Pre-F in enhancing the T cell response to HN component and broadening the overall neutralizing antibody response. The role of T cells will be evaluated in future studies.

Due to mumps outbreaks occurring among individuals twice-vaccinated with MMR, innovation is needed with current mumps vaccines to reduce disease incidence and the burden on public health resources (35, 36). A third dose of mumps-containing vaccine has been recommended by the advisory committee on immunization practices (ACIP) to individuals at risk of contracting mumps in an outbreak setting, and there was a 78% reduced risk of contracting mumps infection after a third dose of MMR compared to individuals who had received two doses of MMR (37, 38). Yet, the response to a third dose can be dampened by preexisting immunity, and increases in neutralization titers are very modest and short-lived (15, 39). The subunit protein vaccine candidates described herein may provide an alternative vaccine modality than the administration of MMR in a mumps outbreak setting.

Multivalent vaccines have been required to protect against multiple serotypes of viruses, for example, for flavivirus vaccines (dengue virus serotypes 1 to 4) (40), influenza vaccines (41), and HPV vaccines (42). The Pre-F/HN chimera, comprising both key targets of neutralizing antibodies on mumps virions is capable of eliciting potent cross-genotype neutralizing responses to mumps genotypes A, G, and H and therefore could be a vaccine candidate for most or all circulating global mumps strains. Combining the structure-based approach with technology improvements in genetic delivery and adjuvantation may provide an effective mumps vaccine that can improve immune titers in recipients and provide protection from recurring mumps outbreaks (34, 43). It remains an important question whether the antibody responses generated from these immunogens could confer protection from disease, as the correlate of protection to mumps infection remains unclear and the role of T cell immunity is not clear although neutralizing antibody titers appear to be associated with protection (14, 17–19). Further preclinical and clinical testing are required to determine the potential for the vaccine candidates described here to contribute to elimination efforts for mumps, either as a booster or a a next-generation mumps vaccine class of primary mumps vaccines for humans.

## Materials and Methods

The design of prefusion-stabilizing mumps F mutations was performed based on the PIV5 F prefusion structures (4GIP and 4WSG) and PIV3 F postfusion (1ZTM) structure, and designs were assessed by negative-stain EM. Proteins were expressed in Expi293 cells transfected with Turbo293 transfection reagent (SPEED BioSystem) and proteins were purified using a His_6_-strepTagII tag and size exclusion chromatography. CB6F1/J mice were immunized to assess the elicitation of neutralizing antibodies by mumps glycoprotein immunogens adjuvanted with Poly I:C. Murine antibodies were isolated by FACS sorting and degenerate primer amplification of heavy and light chains as previously described (23). Mumps genotypes A, G, and H PRNT assays were conducted to determine the potency of sera and monoclonal antibodies. More detailed information is provided in **SI Appendix*, Materials and Methods*.

## Supplementary Material

Appendix 01 (PDF)

## Data Availability

Atomic coordinates for the mumps pre-F protein have been deposited into the PDB under accession number 9DRQ (44). All other study data are included in the article and/or *SI Appendix*.
