# Supplementary material for: Structure-based design of glycoprotein subunit vaccines for mumps
Source: Proc Natl Acad Sci U S A. 2024 Nov 11;121(47):e2404053121. doi: 10.1073/pnas.2404053121 (PMC11588112; doi:10.1073/pnas.2404053121)
Supplement: Supplementary file 1 — Appendix 01 (PDF) [file pnas.2404053121.sapp.pdf]

PNAS

Supporting Information for

Structure-Based Design of Glycoprotein Subunit Vaccines for Mumps

Rebecca J. Loomis[a,1], Yen-Ting Lai[a,1], Sun B. Sowers[b,1], Brian Fisher[a], Alexandrine Derrien-Colemyn[a], David R. Ambrozak[a],Yaroslav Tsybovsky[c], Stephen N. Crooke[b,2], Donald R. Latner[b], Wing-Pui Kong[a], Tracy J. Ruckwardt[a,2], Stanley A. Plotkin[d], Peter D. Kwong[a], John R. Mascola[a], Barney S. Graham[a], Carole J. Hickman[b], Guillaume B. E. Stewart-Jones[a,2]

[a] Vaccine Research Center, National Institutes of Health, Bethesda, MD 20892;
[b] Division of Viral Diseases, National Center for Immunization and Respiratory Diseases, Centers for Disease Control and Prevention, Atlanta, Georgia;
[c] Electron Microscopy Laboratory, Cancer Research Technology Program, Frederick National Laboratory for Cancer Research sponsored by the National Cancer Institute, Frederick, MD 21701;
[d] Department of Pediatrics, University of Pennsylvania, Vaxconsult, 4650 Wismer Rd, Doylestown, PA 18902.

[1] These authors contributed equally.

[2] To whom correspondence should be addressed:
scrooke@cdc.gov (S.N.C.); (404) 718 4003 for neutralization assays.
truckwardt@mail.nih.gov (T.J.R.); (301) 761 7010 for immunization.
gstejones@gmail.com (G.S-J.); (570) 690 7363 for structure-based vaccine design.

This PDF file includes:

Supporting text
Figures S1 to S3
Tables S1 to S5
SI References

Supporting Information Text

Materials and Methods

Structure-based design of prefusion mumps F glycoprotein trimers. Using the homologous model from the PIV-5 prefusion glycoprotein crystal structure (PDB ID 4GIP, 4WSG) we designed 7 disulfide bonds and 3 junctional positions of the C-terminal stem to the coiled coil GCN4 (*SI Appendix,* Fig. S1*A*). F constructs protein expression yields from Expi293 cells were obtained and biophysical and structural characteristics determined to confirm the proportion of prefusion conformation (*SI Appendix,* Fig. S1*B, C*). Using negative stain EM as a readout for conformation, prefusion-stabilized mumps F glycoprotein trimers containing various disulfide-stabilizing designs displayed almost 100% retention of the prefusion conformation (*SI Appendix,* Fig. 1*A*).

Protein expression and purification. Mumps F, HN and Pre-F/HN glycoproteins were expressed by transfection in Expi293 cells (ThermoFisher Scientific) using Turbo293 transfection reagent (SPEED BioSystem) according to the manufacturer's protocol. Transfected cells were incubated in shaker incubators at 120 rpm, 37°C, 9% CO_2 overnight. On the second day, one tenth culture volume of CellBooster medium (ABI scientific) was added to each flask of transfected cells and cell cultures were incubated at 120 rpm, 37°C, 9% CO_2 for an additional 5 days. 6 days post-transfection, cell culture supernatants were harvested and proteins were purified from the supernatants using tandem Ni^{2+} (Roche) and Streptactin (IBA) affinity purification. The C-terminal purification tags were removed by thrombin digestion at room temperature overnight and proteins were further purified by SEC in a Superdex 200 column (GE) in PBS.

Expression screening of mumps F and HN immunogens. Initial assessment of all constructs was performed using a 96-well microplate format for high throughput expression followed by an BLI-based expression evaluation by binding to the StrepTagII by StrepMab Immo (IBA). Briefly, 24 h prior to transfection HEK 293T cells (Thermo Fisher Scientific, MA) were seeded in each well of a 96-well microplate at a density of approximately $2.5x10^5$ cells/ml in expression medium (high glucose DMEM supplemented with 10% ultra-low IgG fetal bovine serum and 1x-non-essential amino acids), and incubated at 37°C, 5% CO_2 for 20 h. Plasmid DNA and Turbo293 transfection reagent (SPEED BioSystem) were mixed and added to the growing cells, and the 96-well plate incubated at 37°C, 5% CO_2. One day post transfection, enriched medium (high glucose DMEM plus 10% ultra-low IgG fetal bovine serum, 2x nonessential amino acids, 1x glutamine) was added to each well, and the 96-well plate was returned to the incubator for continuous culture. Five days post transfection, supernatants with the expressed variants were harvested and tested by BLI for binding to StrepTagII antibody on Octet sensors.

Negative-stain electron microscopy. Proteins, including Pre-F, Post-F and Pre-F-Fab complexes, purified to >95% purity were diluted with 10 mM HEPES, pH 7.0, 150 mM NaCl, adsorbed to a freshly glow-discharged carbon-film grid, washed with the same buffer, and stained with 0.7% uranyl formate. Images were collected at a magnification of 100,000 using SerialEM (1) on an FEI Tecnai T20 microscope equipped with a 2k x 2k Eagle CCD camera and operated at 200 kV. The pixel size was 0.22 nm. Particles were picked using the swarm mode in e2boxer from the EMAN2 software package (2), followed by manual corrections. Reference-free 2D classifications were performed using EMAN2 and SPIDER (3). To determine fractions of prefusion and postfusion molecules, each particle in a dataset was aligned automatically using SPIDER to either a selection of 2D class averages (mumps F and F-HN, Pre-F-Fab complexes). Particles were then assigned to either the prefusion or postfusion fraction based on the highest value of cross-correlation coefficient.

Crystallization, X-ray data collection, structure determination and model building for mumps Pre-F. Mumps Pre-F protein design V206C-A223C, 101KRF-101GGG and 476-GCN4 was expressed in HEK 293T $GnTI^{-/-}$ cells and purified as described above,

deglycosylated with Endo H (NEB), purified by SEC and crystallized in sitting drops from 10mg/ml protein solution in 0.1 M imidazole pH 8.0, 35% PEG1000, 0.2 M calcium acetate. Crystals diffracted in spacegroup R32:H, and diffraction extended to 2.16 Å resolution. Structure solution by molecular replacement with PIV5 Pre-F probe 4GIP followed by rigid body fitting revealed some general architectural homology but with structural variation in many regions; rebuilding to optimize geometry and stereochemistry yielded Rwork/Rfree values of 20.7%/24.6% (*SI Appendix,* Table S1). Diffraction data were processed with the HKL2000 suite (4). The protein polypeptide chain for the mumps Pre-F was re-built using Autobuilder (5) to eliminate any model bias from the PIV5 Pre-F probe. Refinement was carried out with Phenix (6) and Buster (7). Model building was carried out with Coot (8). Carbohydrates were modelled into *F*o-*F*c maps and further refined using *F*o-*F*c, *2F*o-*F*c and feature-enhanced maps (9). The Ramachandran plot as determined by MOLPROBITY (10) showed >89.4% of all residues in favored regions and >99.5% of all residues in allowed regions. Data collection and refinement statistics are shown in *SI Appendix,* Table S1.

Immunogenic characterization of mumps glycoprotein immunogen designs in mice
To assess the effectiveness of recombinant mumps F trimer, mumps HN and Pre-F-HN designs at eliciting neutralizing antibodies, groups of 10 CB6F1/J mice were immunized three times, at weeks 0, 3, and 10 with 10 µg of recombinant mumps F glycoprotein trimer designs combined with 10 µg Poly I:C (Sigma-Aldrich) and 2 weeks after immunization, and monthly thereafter, sera were assessed for heterologous mumps neutralization *in vitro.* Neutralizing antibody titers were determined using a plaque reduction neutralization (PRNT) assay. Neutralizing antibody titers to the JL mumps vaccine strain and wild-type strains (genotype G and H) were determined by the PRNT assay as described previously (11). Sera were heat-inactivated for 30 min and serially diluted 4-fold starting from 1:2 to 1:2,048 and mixed with an equal volume of mumps virus that yielded 40 to 60 plaque-forming units, containing a final dilution ranging from 1:4 to 1:4,096. PRN ID60 titers were determined to be the highest dilution of serum that gave 60% or higher plaque reduction compared with the average number of plaques formed in the absence of serum or monoclonal antibody by using the Kärber formula. Monoclonal antibodies were tested at a range from 0.03125 to $6.1x10^{-5}$ mg/mL and the half maximal inhibitory PRN dose (ID_{50}) were determined.

Probe-based B cell sort from splenocytes of immunized mice
Mumps Pre-F and HN head group plasmids were engineered to contain the AVItag sequence (GLNDIFEAQKIEWHE) upstream of the purification tags, and proteins were purified as described. After expression and purification, the proteins were biotinylated using the BirA biotin-protein ligase reaction kit (Avidity, #BirA500). Biotinylation of the MuV protein probes was confirmed by biolayer interferometry by testing the ability of the biotinylated protein to bind to streptavidin sensors. To conjugate protein probes with the streptavidin-fluorochrome reagents, in a stepwise process, 1/5 of the molar equivalent of the streptavidin-fluorochrome reagent was added to the biotinylated MuV F or HN protein at 20 min intervals until the molar ratio of streptavidin-fluorochrome reagent: biotinylated protein reached 1:1. The incubation was carried out at 4°C with gentle rocking. Streptavidin-allophycocyanin (SA-APC) (Invitrogen) was mixed with biotinylated MuV F or HN protein.

Splenocyte isolation and staining for flow cytometry. Mice were sacrificed with CO_2 and spleens harvested into 5 mL RPMI, 10% FBS, 1% Pen/Strep (1-2 spleens/tube) in gentleMACS tube. Spleens were harvested and mashed using SPLEEN_01 program on gentleMACS machine, strained through a 70 mm strainer and centrifuged at 2000 rpm for 10 min at room temperature. The pellet was resuspended in 5 mL ACK Lysis Buffer/Spleen (2 spleens = 10 mL), lysed for 5 min at room temperature, and an equal volume of RPMI, 10% FBS, 1% Pen/Strep was added, the sample centrifuged at 1800 rpm for 8 min at room temperature. The pellet was resuspended in 10 mL 1X PBS, centrifuged at 1500 rpm for 5 min at room temperature. The pellet was resuspended in 3 mL 1X PBS and transferred to a 5 mL FACS tube, centrifuged, resuspended in 100 mL 1x PBS + 0.125 µL Aqua (Invitrogen) or UV Blue (Invitrogen) and incubated 15-20 min at room temperature. Cells were washed with 1 mL 1x PBS and centrifuged, resuspended in 50:1 F_c

Block:BV Buffer, incubated 2-5 min at room temperature, the antibody mix added (1:1 ratio with the F_c Block + BV Buffer solution) and incubated at 4°C for 20 minutes. Antigen specific B cells were identified with a panel of ligands including fluorescently labeled antibodies for B220, F4/80, Gr-1, CD4, CD8, IgG and IgM. Splenocytes were stained with an antibody cocktail consisting of anti-B220-BV421 (Biolegend), F4/80-BV510 (Biolegend), CD4-BV510 (Biolegend), CD8-BV510 (Biolegend), IgG(1/2/3)-FITC (BD Pharmingen), and IgM-PE-Cy7 (Southern Biotech) with MuV Pre-F-SA-APC. In addition, aqua blue (Invitrogen) was used to exclude dead cells. For titering of probes, the volume was brought up (enough to cover all titration samples : 50 µL / sample) and distributed to dilution tubes. Dilution tubes started with 20 µL antibody in 80 µL BV Buffer and had 8-point, 2x dilutions. Dilutions were incubated with probe with antibody mix from above (50 µL + 50 µL) for 20 min at 4°C. 2 mL FACS Buffer (1x PBS, 0.1% sodium azide) was used to wash cells and they were centrifuged. Pellets were resuspended with 100 µL FACS Buffer + 100 µL 1% PFA in 1X PBS solution.

Isolation of antigen-specific memory B cells by fluorescence activated cell sorting (FACS). Splenocytes were sorted by gating for antigen-specific memory B cells, specifically live IgG+ B cells which were also positive for the MuV Pre-F^-APC probe (live, $B220^+$, $CD4^-$/$CD8^-$/$F4/80^-$/$Gr\text{-}1^-$, IgM^-, IgG^+ and MuV Pre-F^+). 8 x 96-well plates were index sorted using a FACS Aria II (BD Biosciences) interfaced with FacsDiva software (BD Biosciences). Flow cytometry analyses were performed using FlowJo software (Tree Star, Inc.).

Isolation of MuV specific B cell Sequences. Probe positive mouse IgG B cells were sorted individually into 96-well PCR plates and stored at -80°C. All primer sequences used are consistent with those previously described (12, 13) (*SI Appendix,* Table S3). The first strand cDNA for the expressed H and L chain was synthesized using a commercially available Superscript III reverse transcription kit (Invitrogen). The 25 µL reverse transcription reaction mixture consisted of 1X PCR buffer, 1µM of IgG and IgK reverse primers, 1 mM dNTP mix, 0.5% IGEPAL, 7 fM DTT, 1U of RNase inhibitor, 1U of SuperScript III reverse transcriptase (Invitrogen). The reaction was performed as follows; 42°C for 10 min, 25°C for 10 min, 50°C for 60 min, 94°C for 5 min. This cDNA reaction was used for the 1st round PCR of IgG chains using a commercially available Hotstart DNA polymerase kit (Qiagen). The 10 µL reaction mixture consisted of 1X PCR buffer, 0.2 µM of external mouse IgG primers, 0.2 mM dNTP mix, 1 mM MgCl, 0.5U Taq DNA Polymerase and 1 µL of cDNA reaction. The touchdown PCR reaction was performed as follows; 95°C for 5 min, [94°C for 30 sec, 62°C for 30 sec, 72°C for 60 sec > 5x], [94°C for 30 sec, 60C for 30 sec, 72°C for 60 sec > 2x], [94°C for 30 sec, 55°C for 30 sec, 72°C for 60 sec > 2x], [94°C for 30 sec, 52°C for 30 sec, 72°C for 60 sec > 25x], [94°C for 30 sec, 56°C for 30 sec, 72°C for 60 sec > 10x], 72°C for 10 min. This 1st round IgG PCR reaction was used for the 2nd round PCR of IgG chains using a commercially available Hotstart DNA polymerase kit (Qiagen). The 10 µL reaction mixture consisted of 1X PCR buffer, 0.2 µM of internal mouse IgG primers, 0.2 mM dNTP mix, 1X Q solution, 0.5U Taq DNA Polymerase and 0.5 µL of 1st round PCR reaction. The touchdown PCR reaction was performed as follows; 95°C for 5 min, [94°C for 30 sec, 62°C sec for 30 sec, 72°C for 60 sec > 5x], [94°C for 30 sec, 60°C for 30 sec, 72°C for 60 sec > 2x], [94°C for 30 sec, 55°C for 30 sec, 72°C for 60 sec > 2x], [94°C for 30 sec, 52°C for 30 sec, 72°C for 60 sec > 25x], 72°C for 10 min. The same cDNA reaction was also used for the 1st round PCR of IgK chains using a commercially available Hotstart DNA polymerase kit (Qiagen). The 10 µL reaction mixture consisted of 1X PCR buffer, 0.2 µM of external mouse IgK primers, 0.2 mM dNTP mix, 1 mM MgCl, 0.5U Taq DNA Polymerase and 1 µL of cDNA reaction. The PCR reaction was performed as follows; 95°C for 5 min, [94°C for 30 sec, 50°C for 30 sec, 72°C for 55 sec > 50x], 72°C for 10 min. This 1st round IgK PCR reaction was used for the 2nd round PCR of IgK chains using a commercially available Hotstart DNA polymerase kit (Qiagen). The 10 µL reaction mixture consisted of 1X PCR buffer, 0.2 µM of (21) internal mouse IgG primers, 0.2 mM dNTP mix, 1X Q solution, 0.5U Taq DNA Polymerase and 0.5 µL of 1st round PCR reaction. 5 µL of both IgG and IgK reactions were analyzed using DNA electrophoresis and the remaining 5 µL was used for Sanger sequencing (ACTG Inc.) with the respective internal primers for 2nd round reactions. Sequences were analyzed via IMGT/HighV-QUEST and in-house post-processing tools. Selected mouse IgG and IgK chains were cloned into humanized IgH and IgL vectors (GenScript).

Expression of murine mumps specific antibodies. Heavy and light chain plasmids containing the mumps F or HN specific murine immunoglobulin genes were co-transfected in Expi293 cells using Turbo293 transfection reagent (SPEED BioSystem) according to manufacturer's protocol. Cultures were fed with fresh 293FreeStyle media (Life Technologies) 4 h post-transfection and then with HyClone SFM4HEK293 enriched medium (HyClone) containing valproic acid (4 mM final concentration) 24 h after transfection. Cultures were incubated at 37º C for 6 days, and supernatants harvested, clarified by centrifugation and filtration and passed over a protein A affinity column. After a PBS wash and low pH elution, the eluate was pH-neutralized with 1M Tris pH 8.0 and dialyzed against PBS.

Expression of mumps F-specific Fabs in complex with Pre-F. Plasmids for select heavy chains were modified to replace the Fc domain with a His6 tag. These were co-transfected with light chains and the mumps Pre-F plasmid (V206C-A223C with 476-GCN4 and 101KRF-101GGG) with a C-terminal StrepTagII in Expi293 cells using Turbo293 transfection reagent (SPEED BioSystem) according to manufacturer's protocol as described before. The resulting Fab-Pre-F complexes were isolated by Streptactin XT resin (IBA) purification and elution and submitted for negative-stain electron microscopy.

ELISA and competition binding of mumps F-specific antibodies. Binding of F-specific antibodies was evaluated using an ELISA. Wells were coated with Pre-F or Post-F at a concentration of 2 µg/mL in PBS overnight, and the next day blocked (1 hour in TBS/T with 5% dry milk) and washed before incubation with serial 4-fold dilutions of primary antibody. After one hour at 37°C, wells were washed then incubated with the secondary antibody (1:5000 goat anti-mouse HRP in blocking buffer) for an additional hour at 37°C before developing using SureBlue TMB peroxidase. The reaction was stopped with sulfuric acid after 5 minutes. OD450 was graphed and area under the curve calculated using GraphPad Prism. Antibody cross-competition was determined based on biolayer interferometry using a fortéBio Octet Red384 instrument. All assays were performed with agitation set to 1,000 rpm in blocking buffer (1xphosphate-buffered saline (PBS) supplemented with 1% bovine serum albumin (BSA)) to minimize nonspecific interactions. The final volume for all solutions was 60 µL/well. Assays were performed at 30°C in tilted black 384-well plates (Geiger Bio-One). His1K biosensors (forteBio) were equilibrated for >600 sec in 1x PBS prior to loading with his-tagged mumps prefusion F (20 µg/mL in Blocking Buffer (1x PBS, 1% BSA (Sigma)) for 600 sec. Following loading, sensors were incubated for 60 sec in Blocking Buffer prior to incubation with the competitor mAb (20 µg/mL in Blocking Buffer) for 600 sec. Sensors were then incubated in Blocking Buffer for 600 sec before incubation with analyte mAb (20 µg/mL in Blocking Buffer) for 600 sec. Percent competition (PC) of analyte mAbs binding to competitor-bound mumps prefusion F was determined using the equation: PC = 100 – [(analyte mAb binding in the presence of competitor mAb) / (analyte mAb binding in the absence of competitor mAb)] x 100. For all competition experiments, we ensured that antibodies had very slow off rates to mitigate potential artifacts that might have arisen from antibody dissociation. Experiments were performed with IgGs (not Fabs), making use of the intrinsic avidity of bivalent IgGs. A 60% threshold was used to determine competition, consistent with previously published studies (Wang et al, Science 2021, Byrne et al, Nat Comms 2023).

Competition binding of mumps HN specific antibodies

The same protocol as for mumps Pre-F competition binding was used however with some minor modifications. Octet NTA Biosensors (Sartorius) were equilibrated for > 600 s in blocking buffer (1% BSA/PBS) prior to loading with His- tagged Mumps HN monomer (11 mg/mL in blocking buffer) for 900 s. Following loading, sensors were incubated in blocking buffer for 300 s prior to incubation with the competitor mAb (18 mg/mL in blocking buffer) for 900 s. After a further incubation of 300s in blocking buffer, biosensors were incubated with analyte mAb (18 mg/mL in blocking buffer) for 900 s. The percent competition (PC) of analyte mAb binding to competitor-bound mumps HN monomer was determined as described for Pre-F competition binding analyses.

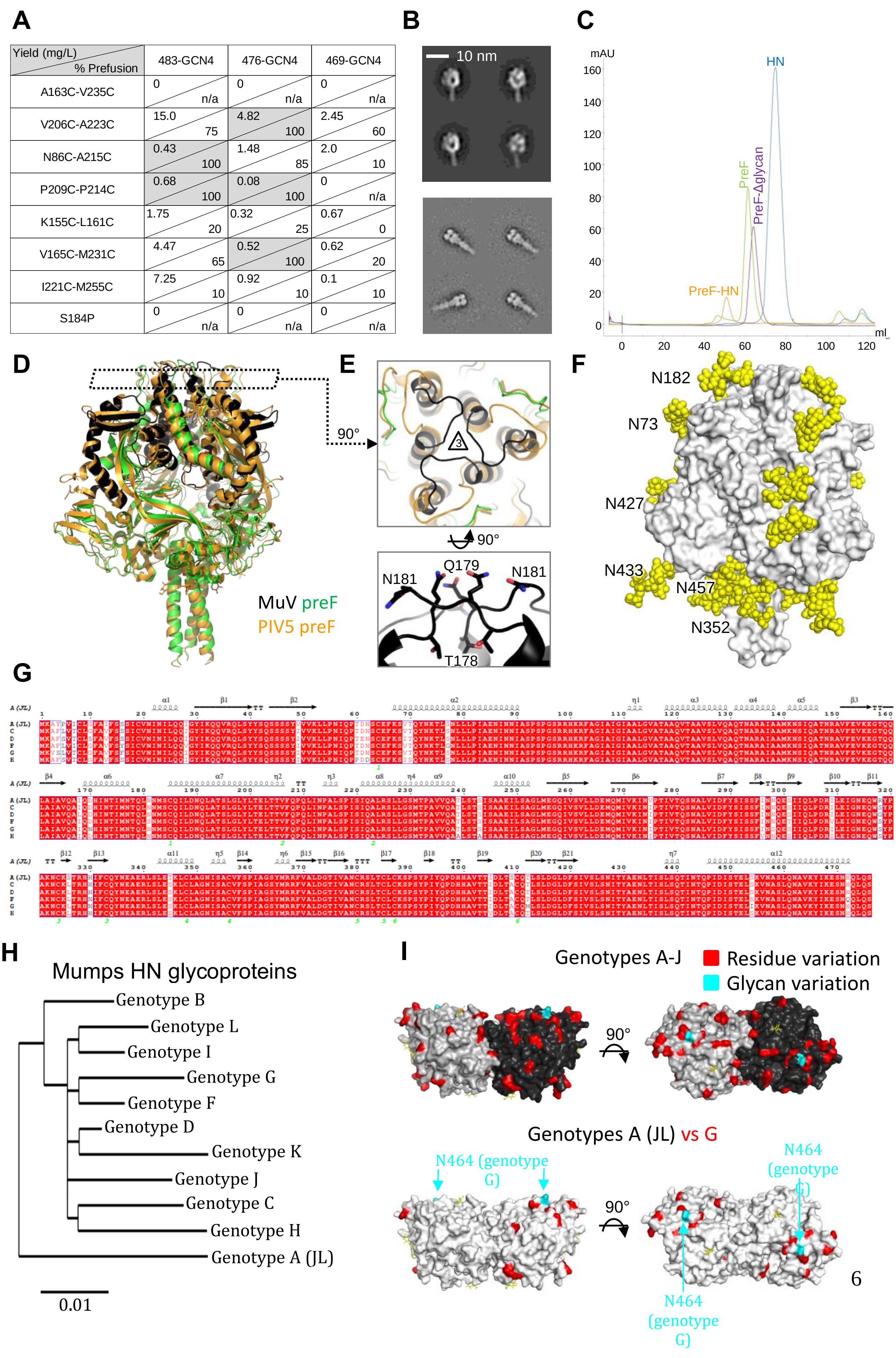

Yield (mg/L) / % Prefusion	483-GCN4	476-GCN4	469-GCN4
A163C-V235C	0 / n/a	0 / n/a	0 / n/a
V206C-A223C	15.0 / 75	4.82 / 100	2.45 / 60
N86C-A215C	0.43 / 100	1.48 / 85	2.0 / 10
P209C-P214C	0.68 / 100	0.08 / 100	0 / n/a
K155C-L161C	1.75 / 20	0.32 / 25	0.67 / 0
V165C-M231C	4.47 / 65	0.52 / 100	0.62 / 20
I221C-M255C	7.25 / 10	0.92 / 10	0.1 / 10
S184P	0 / n/a	0 / n/a	0 / n/a

Fig. S1. Structural and biochemical characterization of disulfide stabilized prefusion mumps F trimer designs, comparing mumps genotype sequences, PIV5 prefusion F structure and glycosylation. (*A*) Structure-based design of prefusion mumps F glycoprotein trimer by systematic screening of disulfides and GCN4 attachment positions, showing both yield and percentage of prefusion relative to postfusion F conformation as determined by negative-stain EM. (*B*) Homogenous prefusion F trimers were observed at high yield with combination of V206C-A223C with 476-GCN4 (upper panel) compared to postfusion F trimers (lower panel). (*C*) S200 gel filtration analysis shows monodispersal of Pre-F (glycosylated and deglycosylated), HN and Pre-F-HN fusion. (*D*) Structural superimposition of mumps prefusion F trimer (colored as in Fig. 1*B*) and the related paramyxovirus PIV5 prefusion F trimer (orange) showing similar overall topology (RMSD = 1.68 Å) despite 49% sequence identity. (*E*) The apical loops of the mumps preF (black) associate in a 'closed cap' assembly, whereas in PIV5 (tan), these loops are splayed apart (top panel). Residues that stabilize the mumps preF apex are T178, T179 and N181 (lower panel). (*F*) The mumps F glycoprotein has 6 *N*-linked glycosylation sites and glycans (yellow) per protomer, totaling 18 per trimer with 2 glycans per protomer (N73 and N182) in the conformationally mobile region and 4 glycans per protomer in the less conformationally variable region (N352, N427, N433 and N457). (*G*) Sequence alignment of Jeryl Lynn vaccine strain (genotype A) with fusion glycoproteins from mumps genotypes C, D, F, G and H. (*H*) Phylogenetic analysis of hemagglutinin-neuraminidase glycoproteins from mumps genotypes A-J. (*I*) Structural mapping of mumps genotype variations on HN, showing A-J genotype variation (top panels) and genotype G versus A (Jeryl Lynn (JL)) (lower panels), showing residue differences (red) and glycan differences (cyan).

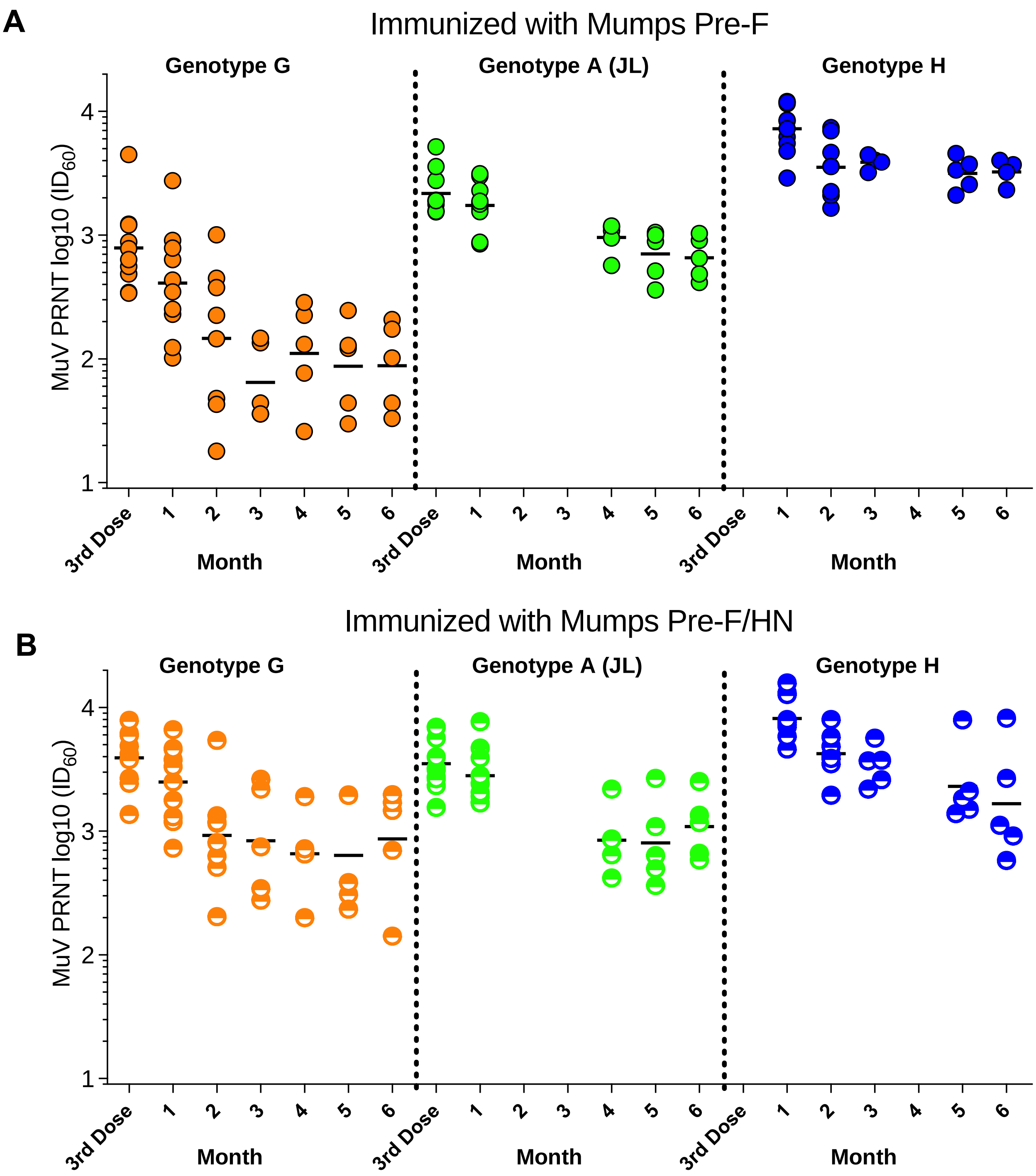
A
Immunized with Mumps Pre-F
Genotype G
Genotype A (JL)
Genotype H
MuV PRNT log10 (ID60)
1
2
3
4
3rd Dose
1
2
3
4
5
6
Month
B
Immunized with Mumps Pre-F/HN
Genotype G
Genotype A (JL)
Genotype H
MuV PRNT log10 (ID60)
1
2
3
4
3rd Dose
1
2
3
4
5
6
Month

Fig. S2. Serological analysis of mumps PreF or mumps PreF-HN immunized mice for specificity and durability of neutralization titers against mumps genotype G, A and H viruses. (*A*) Following three immunizations with 10 mg prefusion F protein with Poly(I:C) with monthly serum sampling and PRNT analyses for six months against genotype G virus (orange), genotype A (Jeryl Lynn) virus (green) and genotype H virus (blue). (*B*) Following three immunizations with 10 mg prefusion F/HN protein with Poly I:C with monthly serum sampling and PRNT analyses for six months against genotype G virus (orange), genotype A (Jeryl Lynn) virus (green) and genotype H virus (blue).

A

Detector	Antibody	Color	Clone	Marker
B515	IgG1	FITC	A85-1	Plasma B cell
B515	IgG2	FITC	R2-40	Plasma B cell
B515	IgG3	FITC	R40-82	Plasma B cell
V450	B220	BV421	RA3-6B2	(CD45R) All stages of B cells
V510	F4/80	BV510	BM8	Dump; Macrophages and DC
V510	CD4	BV510	GK1.5	Dump; CD4 T cells
V510	Gr-1	BV510	R86-8C5	Dump; (Ly6G/Ly6C) Monocytes and Macrophages
V510	CD8	BV510	53-6.7	Dump; CD8 T cells
G780	IgM	PeCy7	1B4B1	Mature B cells
V510	Aqua	BV510		Viability
R670	PreF	APC		Probe

B

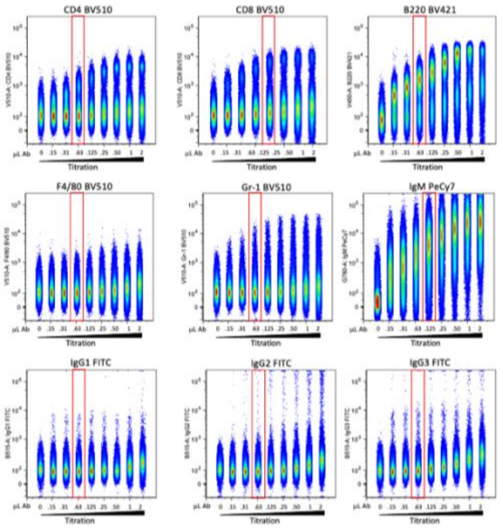

C

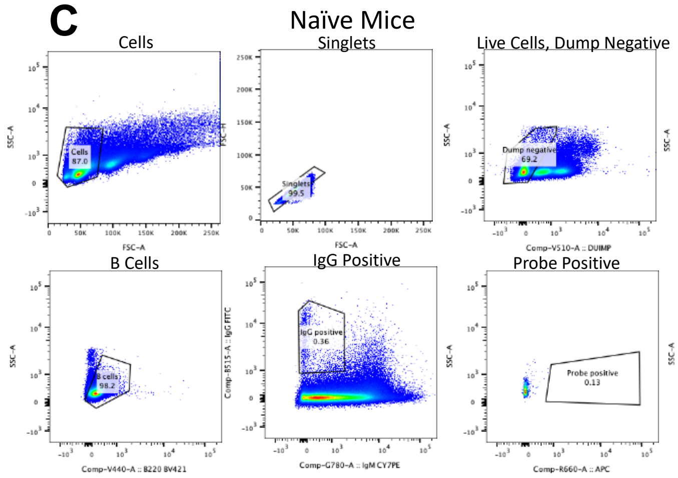

D

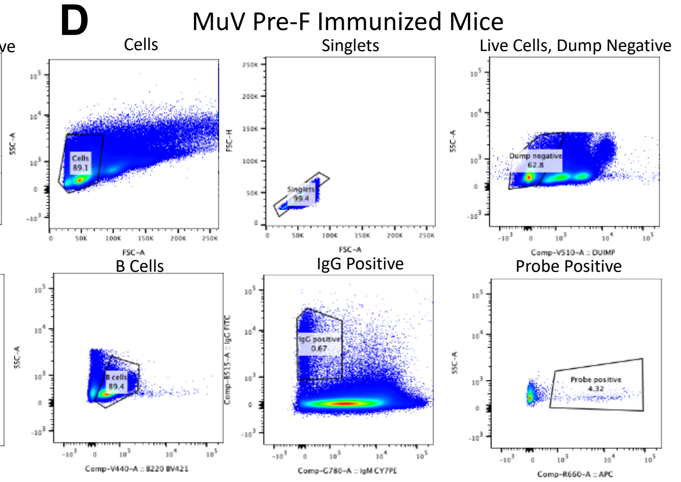

E

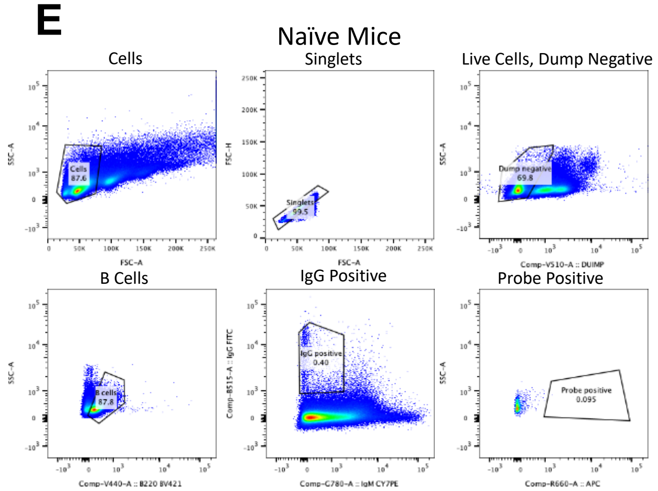

F

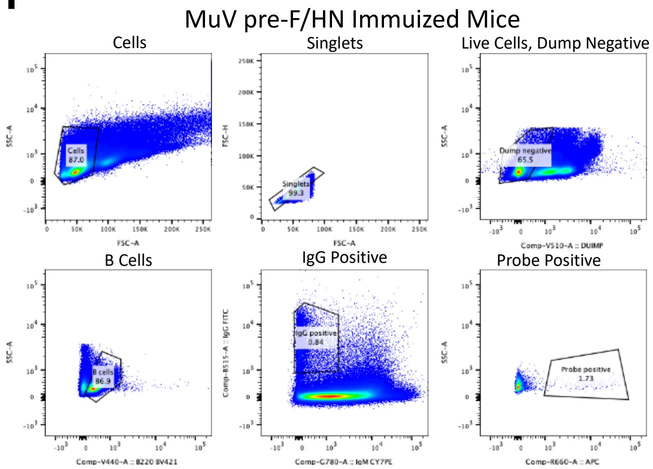

Fig. S3. Probe development, antibody isolation. (*A*) Final antibody panel used for B-cell sort. (*B*) Titration of antibodies used for the sort panel. (*C-F*) Gating strategy for isolation of antigen-specific memory B cells by fluorescence activated cell sorting (FACS). The splenocytes were gated for antigen-specific memory B cells, specifically live IgG+ B cells which were also positive for the (C, D) MuV Pre-F-APC probe (live, B220+, CD4-/CD8-/F4/80-/Gr-1-, IgM-, IgG+ and MuV Pre-F+) or (E, F) MuV HN-BV410 probe (live, B220+, CD4-/CD8-/F4/80-/Gr-1-, IgM-, IgG+ and MuV HN+). Cells index sorted 8 96-well plates using a FACS Aria II (BD Biosciences) interfaced with FacsDiva software (BD Biosciences). Flow cytometry analyses was performed using FlowJo software (Tree Star, Inc.).

Table S1. X-ray data collection and refinement statistics.

	Mumps prefusion F
**Data collection**	
Space group	R32:H
Cell dimensions	
a, *b*, *c* (Å)	74.8, 74.8, 460.9
α, β, γ (°)	90.0, 90.0, 120.0
Resolution (Å)	50-2.16 (2.24-2.16)*#
R_{sym} or R_{merge} (%)	13.4 (165.6);
$I / \sigma I$	18.0 (2.0);
Completeness (%)	99.8 (100.0);
Redundancy	16.6 (16.7);
**Refinement**	
Resolution (Å)	37.41-2.16
No. reflections	27,163
R_{work} / R_{free} (%)	20.71 / 24.57
No. atoms	3,542
Protein	3,368
Ligand/ion	314
Water	40
B-factors	65.82
Protein	64.07
Ligand/ion	113.52
Water	53.63
R.m.s. deviations	
Bond lengths (Å)	0.007
Bond angles (°)	0.894

* Data processing statistics based on the overall resolution cutoff determined as: completeness greater than 50% and I/σI greater than 2.

[#] Statistics for the highest-resolution shell are shown in parentheses.

Table S2. MuV preF and HN Protein Sequences

Mumps Antigen Constructs	
**Antigen Name**	**Antigen sequence**
MuV PreF	MKAFSVTCLSFAVFSSSICVNINILQQIGYIKQQVRQLSYYSQSSSSYIVVKLLPNIQPTDDSCEFKSVTQYNKTLS<br>NLLLPIAENINNIASPSPGSRRHGGGAGIAIGIAALGVATAAQVTAAVSLVQAQTNARAIAAMKNSIQATNR<br>AVFEVKEGTQQLAIAVQAIQDHINTIMNTQLNNMSCQILDNQLATSLGLYLTELTTCFQPQLTNPALSPISIQC<br>LRSLLGSMTPAVVQATLSTSISAAEILSAGLMEGQIISVLLDEMQMIVKINIPTIVTQSNALVIDFYSISSFINNQE<br>SIIQLPDRILEIGNEQWSYPAKNCKLTRHHIFCQYNEAERLSLESKLCLAGNISACVFSPIAGSYMRRFVALDGTI<br>VANCRSLTCLCKSPSYPIYQPDHHAVTTIDLTACQTLSLDGLDFSIVSLSNITYAENLTISLSQTINTQPIDISTELSK<br>VNASLQNAVKYIKESNHQLQSIEDKIEEILSKIYHIENEIARIKKLIGEAP
MuV HN	NIPLVNDLRFINGINKFIIEDYATHDFSIGHPLNMPSFIPTATSPNGCTRIPSFSLGKTHWCYTHNVINANCKDH<br>TSSNQYVSMGILVQTASGYPMFKTLKIQYLSDGLNRKSCSIATVPDGCAMYCYVSTQLETDDYAGSSPPTQKL<br>TLLFYNDTVTERTISPSGLEGNWATLVPGVGSGIYFENKLIFPAYGGVLPNSTLGVKSAREFFRPVNPYNPCSGP<br>QQDLDQRALRSYFPSYFSNRRIQSAFLVCAWNQILVTNCELVVPSSNQTMMGAEGRVLLINNRLLYYQRSTS<br>WWPYELLYEISFTFTNSGPSSVNMSWIPIYSFTRPGSGNCSGENVCPTACVSGVYLDPWPLTPYSHQSGINR<br>NFYFTGALLNSSTTRVNPTLYVSALNNLKVLAPYGTQGLFASYTTTTCFQDTGDASVYCVYIMELASNIVGEFQ<br>ILPVLTRLTIT
MuV PreF-HN	MKAFSVTCLSFAVFSSSICVNINILQQIGYIKQQVRQLSYYSQSSSSYIVVKLLPNIQPTDDSCEFKSVTQYNKTLS<br>NLLLPIAENINNIASPSPGSRRHGGGAGIAIGIAALGVATAAQVTAAVSLVQAQTNARAIAAMKNSIQATNR<br>AVFEVKEGTQQLAIAVQAIQDHINTIMNTQLNNMSCQILDNQLATSLGLYLTELTTCFQPQLTNPALSPISIQC<br>LRSLLGSMTPAVVQATLSTSISAAEILSAGLMEGQIISVLLDEMQMIVKINIPTIVTQSNALVIDFYSISSFINNQE<br>SIIQLPDRILEIGNEQWSYPAKNCKLTRHHIFCQYNEAERLSLESKLCLAGNISACVFSPIAGSYMRRFVALDGTI<br>VANCRSLTCLCKSPSYPIYQPDHHAVTTIDLTACQTLSLDGLDFSIVSLSNITYAENLTISLSQTINTQPIDISTELSK<br>VNASLQNAVKYIKESNHQLQSIEDKIEEILSKIYHIENEIARIKKLIGEAPGSGGGGGGNIPLVNDLRFINGINKFII<br>EDYATHDFSIGHPLNMPSFIPTATSPNGCTRIPSFSLGKTHWCYTHNVINANCKDHTSSNQYVSMGILVQTAS<br>GYPMFKTLKIQYLSDGLNRKSCSIATVPDGCAMYCYVSTQLETDDYAGSSPPTQKLTLLFYNDTVTERTISPSGL<br>EGNWATLVPGVGSGIYFENKLIFPAYGGVLPNSTLGVKSAREFFRPVNPYNPCSGPQQDLDQRALRSYFPSYF<br>SNRRIQSAFLVCAWNQILVTNCELVVPSSNQTMMGAEGRVLLINNRLLYYQRSTSWWPYELLYEISFTFTNS<br>GPSSVNMSWIPIYSFTRPGSGNCSGENVCPTACVSGVYLDPWPLTPYSHQSGINRNFYFTGALLNSSTTRVNP<br>TLYVSALNNLKVLAPYGTQGLFASYTTTTCFQDTGDASVYCVYIMELASNIVGEFQILPVLTRLTIT

Table S3. Mouse BCR and Sequencing Primers.

1st round IgH		1st round IgK	
VH1 Ext	agRtYcagctgcaRcagtct	Vkappa1 Ext	tgatgacccaRactccact
VH1-2 Ext	aggtccaactgcagcagcc	Vkappa2 Ext	gcttgtgctctggatccc
VH2 Ext	tctgcctggtgacWttccca	Vkappa3 Ext	ctgctgctctgggttcc
VH3 Ext	gtgcagcttcaggagtcag	Vkappa4 Ext	cagcttcctgctaatcagtg
VH4 Ext	gaggtgaagcttctcgagtc	Vkappa5 Ext	ctcagatccttggacttHtg
VH5 Ext	gaagtgaagctggtggagtc	Vkappa6 Ext	tggagtcacagacYcagg
VH6 Ext	atgKacttgggactgaRctgt	Vkappa7 Ext	tggagtttcagacccagg
VH7 Ext	cagtgtgaggtgaagctggt	Vkappa8 Ext	ctgctMtgggtatctggt
VH8 Ext	ccaggttactctgaaagagtc	Vkappa9 Ext	cWtcttgttgctctggtttc
VH9 Ext	tgtggaccttgctattcctga	Vkappa10 Ext	gatgtcctctgctcagttc
VH10 Ext	tgttggggctgaagtgggttt	Vkappa11 Ext	cctgctgagttccttggg
VH11 Ext	atggagtgggaactgagctta	Vkappa12 Ext	ctgctgctgtggcttaca
VH12 Ext	agcttcaggagtcaggacc	Vkappa13 Ext	ccttctcaacttctgctct
VH13 Ext	caggtgcagcttgtagagac	Vkappa14 Ext	agggcccYtgctcagttt
VH14 Ext	atgcagctgggtcatcttctt	Vkappa15 Ext	atgagggtccttgctgag
VH15 Ext	gactggatttggatcacKctc	Vkappa16 Ext	gaggttccaggttcaggt
VH16 Ext	tggagtttggacttagttggg	Vkappa17 Ext	ccatgaccatgYtctcact
Cgamma Ext	agggaaataRcccttgaccag	Vkappa18 Ext	atggaaactccagcttcattt
Cgamma-2 Ext	agggaagtagcctttgacaag	Vkappa19 Ext	atgagaccgtctattcagtt
		Ckappa Ext	gcacctccagatgttaactg
2nd round IgH		2nd round IgK	
VH1 Int	gaRgatRtcctgYaaggcttc	Vkappa1 Int	cctgtcagtcttggagatca
VH1-2 Int	aRgBtgtcctgcaagRcttc	Vkappa1-2 Int	tttgtcggttaccattggacaa
VH2 Int	tgcagctgaagSagtcagga	Vkappa2 Int	SRgatattgtgatgacgcagg
VH3 Int	aaccttctcagWcactgtcc	Vkappa3 Int	attgtgctgacccaatctcc
VH4 Int	ggaggtggcctggtgcag	Vkappa4 Int	aWtgtKctcacccagtctcc
VH5 Int	agcctggagggtccctgaa	Vkappa5 Int	gtctccagccaccctgtc
VH6 Int	gaggagtctggaggaggctt	Vkappa6 Int	tgatgacccagtctcMcaaat
VH7 Int	tctggaggaggcttggtaca	Vkappa7 Int	gcctgtgcagacattgtgat
VH8 Int	ctgggatattgcagccctcc	Vkappa8 Int	cctgtggggacattgtgatg
VH9 Int	acagatccagttggtgcagt	Vkappa9 Int	acatccRgatgacYcagtct
VH10 Int	aggtgtgcattgtgaggtgc	Vkappa10 Int	ccagatgtgatatccagatg
VH11 Int	gaagtgcagctgttggagac	Vkappa11 Int	gccagatgtgatgtYcaaatg
VH12 Int	cctggtgaaaccctcacag	Vkappa12 Int	atccagatgactcagtctcc
VH13 Int	aggcttggtgaggcctgga	Vkappa13 Int	cctgatatgtgacatccRVat
VH14 Int	gaggttcagctgcagcagt	Vkappa14 Int	Magatgacccagtctccatc
VH15 Int	caatcccaggttcacctacaa	Vkappa15 Int	tgagatgtgacatccagatga
VH16 Int	gtgaggtgcagctggtgga	Vkappa16 Int	ccagtgtgatgtccagataac
Cgamma Int	ggccagtggatagacHgatg	Vkappa17 Int	acaactgtgacccagtctcc
Cgamma-2 Int	cagggaccaagggatagaca	Vkappa18 Int	acacaggctccagcttctct
		Vkappa19 Int	gtgctcagtgtgacatccag
		Ckappa Int	gatggtgggaagatggatac

Chain	Sequencing Primers		
IgH-1	Cgamma Int		ggccagtggatagacHgatg
IgH-2	Cgamma-2 Int		cagggaccaagggatagaca
IgK	Ckappa Int		gatggtgggaagatggatac

Table S4. Mumps Pre-F Antibody VH/VL Amino Acid Sequences

Mumps Pre-F Antibody VH/VL Amino Acid Sequences	
**Antibody Name**	**Amino Acid Sequence**
H5 Heavy Chain	EVQLQQSGPELEKPGASVKISCKASGYSFTGYNMNWVKKSNGKSLEWIGNIDPYYGGTSYNQKFKGKATLTVDKSSSTAYMQLKSLTSEDSAVYYCARGSLPFFDYWGQGTTLTVSS
H5 Light Chain	DIVMTQSQKFMSTSVGDRVSITCKASQNVGTAVAWYQQKPGQSPKLLIYSASNRYTGVPDRFTGSGSGTDFTLTISNMQSEDLADYFCQQYSSYPHTFGGGTKLEIK
F3 Heavy Chain	EIQLQQSGPELEKPGASVKISCKASGYSFTDYNIHWVRQSNGKSLEWIGNIDTYYGGTSYNQRFMGKATLTVDKSSSTAYMQFKSLTSEDSAVYFCARWLLRGLDYWGQGTTLTVSS
F3 Light Chain	DIVMTQSQKFMSTSVGDRVSITCKASQNVGTAVAWYQQKPGQSPELLISSASNRYTGVPDRFTGSGSGTDFTLTINNVQSEDLADYFCQQYSTYPLTFGTGTKLELK
B4 Heavy Chain	EVQLQQSGAELVKPGASVKMSCKASGYTFTSYWMHWVKQRPGRGLEWIGRIDPNSGGTKYNEKFKSKATLTVDKPSSTAYMQLSSLTSEDSAVYYCARSRIFDAYYGYTMDYWGQGTSVTVSS
B4 Light Chain	NIVLAQSPASLAVSLGQRATISCRASESVDSYGNSFMHWYQQKPGQPPKLLIYLASNLESGVPARFSGSGSRTDFTLTIDPVEADDAATYYCQQNNEDPRTFGGGTKLEIK
C4 Heavy Chain	EVQLQQSGAELVMPGASVKMSCKASGYTFTNYWIQWMKQRPGQGLEWIGEIDPSDSYTNYNQKFKGKSTLSVDKSSSTAYIQLSSLTSEDSAVYYCAREELYSNYDGFDFWGQGTTLTVSS
C4 Light Chain	DIVMTQSPSSLALSVGQTVTMNCESSQSLLNTNNQINYLAWYQQKPGQSPKLLVYFASLRESGVPDRFMGSGSGTDFTLTISSVQAEDLADYFCQQHYTTPYTFGGGTKLEIK
H4 Heavy Chain	EVQLQQSGAELVRSGASVKLSCTASGFNIEDFYIHWVRQRPEQGLEWIGWIDPENSLTEYSPKFLDRATVTADTSSNTAYLQLSSLTSEDTAVYYCNPKTPYYYVRTSYYFDYWGQGTTLTVSS
H4 Light Chain	DIVMTQSPSSLAMSVGQKVTMSCKSSQSLLNNNNQENYLAWYQQKPGQSPKLLVYFASTRESGVPDRFIGRGSGTDFTLTISSVQAEDLADYFCQQHYSTPFTFGSGTKLEIK
F1 Heavy Chain	DLQLKQSGPGLVKPSQSLSLTCSVTGSSIASGYYWNWIRQFPGNKLEWMGYITYDGKNDYNPSLKDRISITRDTTKNQFFLMLTSVTTEDTATYYCARDNFYFDYWGQGTALTVSS
F1 Light Chain	DIVMTQSPSSLAMSVGQKVTMNCKSSQSLLNTNNQENYLAWYQQKPGQSPKLLVYLASTRESGVPDRFIGRGSGTDFSLTIYSVQAEDLADYFCQQHYRTPFTFGSGTKLEIK
E2 Heavy Chain	DVQLQESGPGLVKPSQSLSLSCSVTGYSITSGYYWNWIRQFPGNKLEWMGYITYDGNNDYNPSLKNRISITRDTSKNQFFLKLNSVTTEDTATYYCVRDNFYFDYWGQGTTLTVSS
E2 Light Chain	DIVMTQSPSSLAMSVGQKVTMSCKSSQSLLNSNNQENYLAWYQQKPGQSPKLLVYLASTRESGVPNRFIGSGSGTDFTLTIISVQAEDLADYFCQQHYSTPFTFGSGTKLEIK
C5 Heavy Chain	EVQLQQSGPELVKPGPSVKISCKTSGYSFTYYYINWVKQSPEKSLEWIGEIDPNTDYTTYNQKFKAKATLTVDKSSSTAYMQLKSLTSEDSAVYYCVRSGLLDYAMDYWGQGTSVTVSS
C5 Light Chain	DIVMTQSPSSLSVSAGEKVTMSCKSSQSLLNTGSQKNYLAWYQQKPGQPPKLLIYGASIRDSGVPDRFTGSGSGTDFTLTIRGVQAEDLAVYYCQNDHSYPFTFGPGTKLEIK
B2 Heavy Chain	DVQLKQSGPGLVKPSQSLSLTCSVTGYSITSGYYWHWIRQFPGNKLEWMDYISYDGSSDYNPSLRNRISITRDTSRNQFFLKLDSVTPDDTATYYCARGGIHYYGRGYYKYFDVWGTGTTVTVSS
B2 Light Chain	DIQMNQSPSSLSASLGDTITITCHASQNINIWLSWYQQKPGNIPKLLIYKASNLHKGVPSRFSGSGSGTGFTLTISSLQPEDIATYYCQQGQSFPLTFGGGTKLEIK
C2 Heavy Chain	DVQLKQSGPGLVKPSQSLSLTCSVTGYSITSGYYWHWIRQFPGNKLEWMDYISYDGSSDYNPSLRNRISITRDTSKNQFFLNLDSVTTEDTATYFCARGGIHYYGRGYYKYFDVWGTGTTVTVSS
C2 Light Chain	DIQMTQSPSSLSASLGDPITITCHASQNINVWLSWYQQKPGNIPKLLIYKASILRTGVPSRFSGSGSGTGFTLTISSLQAEDIATYYCQQGQSFPLTFGGGTKLEIK
E6 Heavy Chain	EVQLQQSGPELVKPGASVKMSCKASGYTFTRCFMQWVKQKPGQGLEWIGYINPYNDGTKYNEKFKGKATLTSDKSSSTAYMDLSSLTSEDSAVYYCARSPYTFRYDMGYAMDYWGQGTSVTVSS
E6 Light Chain	NIVLTQSPASLAVSLGQRATISCRASESVDSYGNSFMHWYQQKPGQPPKLLIYLASSLESGVPARFSGTGSRTDYTLIIDPVEADDVATYYCQQNNEDPRTFGGGTKLEIK
G2 Heavy Chain	DVQLKQSGPGLVKPSQSLSLTCSVTGYSITSDYAWNWIRQFPGNELEWMGYITYSGSANYNPSLKSRISITRDTSKNQFFLQLNSVTTEDTATYYCASNMITYFYPLDHWGQGTSVTVSS
G2 Light Chain	DIVMTQSHKFMSTSIGDRVTITCKASQDVSNAVAWYQQKPGQSPKLLIYWASTRHTGVPDRFTGSGSGTDYTLTISSVQAEDLALYYCHQHYNTPWTFGGGTKLEIK
H3 Heavy Chain	EVQLQQSGAEVMKPGASVNLSCKAIGNTFTGYWIEWVKQRPGHGLEWIGEILPGSGNTKYSEKFKGKATFTAETSSNTAYMQLNSLTTEDSAIYYCAYGSSFPPMNHWGQGTSVTVSS
H3 Light Chain	DIVMTQSQKFMSTSVGDRVSITCKASQNVRTAVAWYQHKPGQSPKALVYLASNRHTGVPDRFTGSGSGTDFTLTIRNVQSEDLADYFCLQHWNYPWTFGGGTKLEIK
B6 Heavy Chain	EVQLQQSGPELVRPGASVKMSCKASGYTFTDYNMHWVKQSHGKSLEWIGYINPNQAGANYNQKFKGRAALTVDKSSSTAYLELRSLTSEDSAVYYCGREEVWAMDYWGQGTSVTVSS
B6 Light Chain	DIQMTQSPASLSASVGETVTITCRASGNIHNYLAWYQQKQGKSPQLLISNAKTLADGVPSRFSGSGSGTQYSLRINSLQPEDFGSYFCQHFWNLPWTFGGGTKLEIK

Table S5. Mumps HN Antibody VH/VL Amino Acid Sequences

Mumps HN Antibody VH/VL Amino Acid Sequences	
**Antibody Name**	**Amino Acid Sequence**
G6 Heavy Chain	QIQLVQSGPELKKPGETVKISCKASGYTFTPYGLSWVKQAPGKVLKWMGWINTSSGVPTYADDFKGRFAFSLETSVSTAYLQINNLKNEDTATYFCARISVSFYYGLYFDYWGQGTTLTVSS
G6 Light Chain	DIQMTQSPSSMSVSLGDTVSITCHASQGIANNIGWLQQKPGKSFKGLLYHGSTLEDGVPSRFSGSGSGAAYSLTISSLESEDFADYYCIQYAQLPYTFGGGTKLEIK
C7 Heavy Chain	QITQKSEGSGILQPSQTLSLTCSFSGFSLNISGMGVGWIRQPSGKGLEWLAHIWWDDVKRYNPALKSRLTISKDTSSRQVFLKIASVDTADTATYYCVRIDNSYAMDYWGQGTSVTVSS
C7 Light Chain	DIVLTQSPASLAVSLGQRATISCRASKSVSTSGYSYVHWFQQKPGQPPKLLIYLASNLESGVPARFSGSGSGTDFTLNIHPVEEEDAATYYCQHSRELPWTFGGGTKLEIK
E7 Heavy Chain	EVKLVESGGGLVQPGGSLRLSCATSGFTFTDYYMSWVRQTPGKALEWLGFIRNKPNGYTTEYNASVKGRFTISRDNSRSVLYLQMNVLRAEDSATYFCARVPHDYGFFDYWGQGTTLTVSS
E7 Light Chain	DVLMTQSPLSLPVSLGDQASISCRSSQSIEHINGHTYLEWYLQKPGQSPKVLIYKVSNRFSGVPDRFSGSGSGTDFTLRISRVEAEDLGVYYCFQASHLPYTFGGGTKLEIK
C9 Heavy Chain	EVKLLQSGGGLVQPGGSLKLSCAASGIDFSRYWMGWVRRAPGKGLEWIGEINPDSSTINYAPSLKDKFIISRDNAKNTLYLQMIKVRSEDTALYYCARYWADWFFDVWGTGTTVTVSS
C9 Light Chain	DIQMTQSSSYLSVSLGGRVTITCTASDHIDNWLAWYQQKPGNAPRLLLSGATSLDTGVPSRFSGSGSGKDFTLSITSLQTEDVATYYCQQYWSTPYTFGGGTKLEIK
D9 Heavy Chain	DVQLQESGPGLVKPSQSLSLTCSVTGFSITSGYDWNWIRQFPGNKLEWMGYISYDGSTKYNPSLKNRISITRDTSKNQFFLRLNSVTAEDTATYYCAREAYDGFHYCAMDFWGQGTSVTVSS
D9 Light Chain	DVVVTQTPLSLPVSLGDQVSISCRSSQSLANNYGNTYLSWYLHKPGQSPQLLIYGISNRFSGVPDRFSGSGSGTDFTLKISTIKPEDLGMYYCLQGTHQPWTFGGGTKLEIK
G9 Heavy Chain	EVQLQQSGPEQVRTGASVKISCKASGYFFTGYYLHWVRQSHGKSLEWIGYIRSYNGATKYNQKFEGKTTFTVDTSSSTAYMQINSLTSEDSAVYYCAKDEDSGYAMDYWGQGTSVTVSS
G9 Light Chain	DIVMTQSHKFMSTLVGDRVAITCKASQDVSSAVAWYQQKPGQSPKLLIYSASYRFTGVPDRFTGSGSGTDFTFTITTVQTEDLAIYFCQQHYSLPWTFGGGTKLEIK
E10 Heavy Chain	EVQLQQSGAELVRPGSSVKLSCKASGYTFTWYWIDWMKQRPGQGLEWIGNIYPSDSEVQYNQKFKDRATLTLDKSSTTAYMQLSSLTSEDSAVYYCARRLYDYFALDFWGQGTSVTVSS
E10 Light Chain	DIVMTQAAFSNPVTLGTSASISCRSSKSLQHSNGITYLYWYLQKPGQSPQLLIYQMSKFASGVPDRFSSSGSGTDFTLRISRVEAEDVGVYYCAQNLELPWTFGGGTKLEIK
F10 Heavy Chain	EVQLQQSGAELVRPGSSVKLSCKASGYTFTWYWIDWLKQRPGQGLEWIGNIYPSDSAVQYNQNFKDRATLTLDTSSTTAYLHLSSLTSEDSAVYYCARRIYDYFALDFWGQGTSVTVSS
F10 Light Chain	DIVMTQAAFSHPVTLGTSASISCRSSKSLQHSNGITFLYWYLQKPGQSPQLLIYQMSKLASGIPDRFSSSGSGTDFTLRISRVEAEDVGVYYCAQNLELPWTFGGGTKLEIK
B11 Heavy Chain	EVQLQQSGAEPVRPGTSVKFSCKASGYTLTDYWMHWVKQRPGQGLDWIGVIDPSDSYTTYNQKFKGKATLTVDKSSSTAYMQLSSLTSEDSAVYYCARGPTLYYFDYWGQGTTLTVSS
B11 Light Chain	DIVMTQPQKFMSTSVGDRVSVTCKASQNVGTNVVWCQQKPGQSPKALIYSASYLYSGVPDRFTGTGSGTDFTLTISNVQSEDLAEYFCQQYNNYPLTFGAGTKLELK
D11 Heavy Chain	EVQLQQSGAELVRPGTSVKLSCKTSGYTFISYWIHWVKQRPGQGLEWIGVIDPSDSYTNYNQNFKGKVTLTVDTSSSTAYMQLSSLTSEDSAVYYCARFDYDDYWGQGTTLTVSS
D11 Light Chain	QIVLTQSPAIMSASPGEKVTMTCSASSSVTYMQWYKQKSGTSPKRWIYDTSKLASGVPARFSGSGSGTSYSLTISSMEAEDAATYYCQQWNTNPWTFGGGTKLEIK
F11 Heavy Chain	EVQLQQSGPELVKPGASVKISCKASGYTFTDYYMNWVKQSHGKSLEWVGDINPNNGVTRYNQNFKGKGTMTVDKSSRTAYMEFRSLTSEDSAVYYCGRLGYYGKYKDGMDYWGQGTSVTVSS
F11 Light Chain	DIQMTQSPASLSVSVGETVTITCRASDNIYSNLAWYQQRQGKSPQLLVYAATNLLDGVPSRFSGSGSGTHYSLKINSLQSEDFGTYYCQHFWGTPFTFGSGTKLEIK
G11 Heavy Chain	EVQLQQSGPELVKPGASVKMSCKASGYTFTEYYMKWVKQSHGKSLEWIGDINPNNGDAFYNQKFKGKATLTVDKSSSTAYMQLNSLTSEDSAVYYCTRPDFYYDWYFDVWGAGTTVTVSS
G11 Light Chain	QIVLTQSPALMSASPGEKVTISCSVSSSVSYIYWYQQKPGSSPKPWIYRTSNLASGVPARFSGSGSGTSYFLTISSMEAEDAATYYCQQIQTYPPMYTFGGGTKLEIK
H11 Heavy Chain	EVQVQQSGPELVKPGASVKMSCKASGYTFTDYYMKWVKQSHGKSLEWIGDINPNNGDTFYNQKFKGKATLTVDKSSSIAYMQLNSLTSEDSAVYYCTRPDYYYDWYFDVWGAGTTVTVSS
H11 Light Chain	QCQIVLTQSPAIMSASPGEKVTISCSASSSVSYMYWYQQKPGSSPKPWIYRTSNLASGVPARFSGSGSGTSYSLTISNMEAEDAATYYCQQYQTYPPMYTFGGGTKLEIK
A12 Heavy Chain	EVQLQQSGPELVKPGTSVKISCKSSGYTFTDYALDWVKQSHGESLEWIGDINPDVGGVVLNQKFMGKATLTVDKSSSTAFMELRSLTSEDTAVYYCARRDYDYAGKGFDYWGQGTTLTVSS
A12 Light Chain	DIVMTQSPSSLAVSAGEKVTLNCKSSQSLLYSSSQKNYLAWYQQKPGQSPKLLIYWASTRESGVPDRFAGSGSGTDFTLTISNVQVEDLALYYCHQYLSSWTFGGGTKLEIK
B12 Heavy Chain	EVQLQQSGPELVKPGASVKIPCKASGYTFTEYNMDWVKQSHGKSLEWIGDINPNNGDTIYNQKFKGKATLTVDKASSTAYMELRSLTSEDTAVYYCARGTLYGSNYYFVMDYWGQGTSVTVSS
B12 Light Chain	DIQITQSPASLSASVGETVTITCGASENIYGALNWFQRKQGKSPQLLIYGATTLADGMSSRFSGNGSGRQYSLKISGLHPDDVATYYCQNVLSIPWTFGGGTKLEIK

SI References

1. Mastronarde D.N., et al., Automated electron microscope tomography using robust prediction of specimen movements. *J Struct Biol* **152**(1):36-51 (2005).
2. Tang G*, et al.* EMAN2: an extensible image processing suite for electron microscopy. *J Struct Biol* **157**(1):38-46 (2007).
3. Frank J*,* et al. SPIDER and WEB: processing and visualization of images in 3D electron microscopy and related fields. *J Struct Biol* **116**(1):190-199 (1996).
4. Otwinowski, Z., et al., Processing of X-ray diffraction data collected in oscillation mode. *Methods Enzymol* **276**, 307-326 (1997).
5. Terwilliger, T.C., et al., Iterative model building, structure refinement and density modification with the PHENIX AutoBuild wizard. *Acta Crystallogr D Biol Crystallogr* **64**, 61-69 (2008).
6. Adams, P.D., et al., Recent developments in the PHENIX software for automated crystallographic structure determination. *J Synchrotron Radiat* **11**, 53-55 (2004).
7. Bricogne, G.B., et al., BUSTER version 2.10.0. Cambridge, UK: Global Phasing Ltd. (2011)
8. Emsley, P., and Cowtan, K. et al., Coot: model-building tools for molecular graphics. *Acta Crystallogr D Biol Crystallogr* **60**, 2126-2132 (2004).
9. Afonine, P.V., et al., FEM: feature-enhanced map. *Acta Crystallogr D Biol Crystallogr* **71**, 646-666 (2015).
10. Davis, I.W., et al., MOLPROBITY: structure validation and all-atom contact analysis for nucleic acids and their complexes. *Nucleic Acids Res* **32**, W615-619 (2004).
11. Rasheed, M. A. et al. Decreased humoral immunity to mumps in young adults immunized with MMR vaccine in childhood. *Proc Natl Acad Sci U S A* **116**(38):19071-19076 (2019).
12. Loomis, R. J. et al., Structure-Based Design of Nipah Virus Vaccines: A Generalizable Approach to Paramyxovirus Immunogen Development. *Front Immunol* 11:**842** (2020).
13. Rohatgi, S et al., Systematic design and testing of nested (RT-)PCR primers for specific amplification of mouse rearranged/expressed immunoglobulin variable region genes from small number of B cells. J Immunol Methods **339**(2):205-19 (2008)
